# Shaoyao gancao decoction induces autophagy by modulating miR-21 and inhibiting the AKT/mTOR signaling pathway in adenomyosis-derived ectopic endometrial stromal cells

**DOI:** 10.3389/fphar.2025.1665911

**Published:** 2025-11-27

**Authors:** Huijie Lai, Jinjin Jia, Zijun Kuang, Jie Lu, Yuyan Zeng

**Affiliations:** 1 The Second Clinical College of Guangzhou University of Chinese Medicine, Guangzhou, China; 2 Department of Traditional Chinese Medicine, Qinghai Unversity Medical College, Xining, China; 3 The First People’s Hospital of Foshan City, Foshan, China; 4 Department of Gynecology, The Second Affiliated Hospital of Guangzhou University of Chinese Medicine, Guangzhou, China

**Keywords:** shaoyao gancao decoction, adenomyosis, autophagy, miR-21, Akt/mTOR pathway

## Abstract

**Ethnopharmacological relevance:**

Shaoyao Gancao Decoction (SGD) is a renowned traditional Chinese medicinal formulation widely utilized in Asia. It is particularly effective in treating spastic, painful, and inflammatory conditions, especially gynecological pain disorders. However, the underlying mechanisms of its action remain largely unexplored.

**Aims of the study:**

To investigate the potential molecular mechanism by which SGD inhibits autophagy and apoptosis in ectopic endometrial stromal cells (EESCs), thereby elucidating the pathway responsible for its anti-adenomyosis effects.

**Materials and methods:**

Four types of tissues were collected for detection, including AM ectopic endometrium, AM eutopic endometrium, normal myometrium, and normal endometrium. Meanwhile, EESCs were cultured *in vitro* and divided into three groups: blank control group, blank serum group, and SGD-containing serum group. Autophagy in EESCs after microRNA-21 (miR-21) transfection was observed using a transmission electron microscope (TEM). Apoptosis of EESCs was detected by flow cytometry (FCM). A quantitative real-time polymerase chain reaction (qRT-PCR) assay was adopted to evaluate the expressions of miR-21 and phosphatase and tensin homolog (PTEN). For the expression of proteins related to the relevant signaling pathway, autophagy, and apoptosis, the Western blot technique was employed.

**Results:**

In ectopic endometria of AM, there was a significant increase in miR-21 mRNA expression as well as heightened activity within the phosphatidylinositol 3-kinase/protein kinase B/mammalian target of rapamycin (PI3K/AKT/mTOR) signaling pathway. Conversely, PTEN expression was found to be decreased. Additionally, autophagy-related protein levels were diminished while anti-apoptotic protein expression was markedly elevated (*P* < 0.01). Results from cell experiments indicated that treatment with SGD downregulated miR-21 mRNA expression along with p-AKT and p-mTOR protein levels (*P* < 0.01), while up-regulating PTEN mRNA expression (*P* < 0.01) in EESCs. Simultaneously, the expression of autophagy-related proteins was increased.

**Conclusion:**

The progression of AM is associated with autophagy inhibition in ectopic lesions. SGD can exert autophagy-promoting and pro-apoptotic effects in EESCs, and this process occurs by inhibiting the AKT/mTOR signaling pathway with miR-21 as the target. These findings unveil the potential novel molecular mechanisms from the perspective of microRNA by which SGD suppresses the growth of EESCs.

## 1 Introduction

Adenomyosis (AM) is a prevalent gynecological condition characterized by the proliferation of active endometrial tissue that invades the uterine muscle layer, accompanied by hyperplasia and fibrosis of the surrounding myometrial tissue (Bulun et al., 2023). The clinical presentations of AM may vary. Pain is one of the primary symptoms, which may include dysmenorrhea, chronic pelvic pain, and dyspareunia; these conditions can profoundly impact both the physical and psychological wellbeing of patients (Gruber and Mechsner, 2021). The management of AM presents considerable challenges, as conservative therapies are often associated with high recurrence rates and insufficient symptom resolution. Hysterectomy remains the most effective treatment for AM; however, it is not an acceptable option for many patients (Osada, 2018; Struble et al., 2016).

AM, although benign in nature, exhibits traits typically seen in malignant conditions, including an enhanced ability to invade surrounding tissues, metastatic properties, angiogenesis, and the capacity to evade apoptosis (Zhang H. et al., 2024). During the process of tumor proliferation and migration, the PI3K/AKT/mTOR signaling pathway is often activated, while the tumor suppressor gene PTEN can inhibit the activation of the PI3K/AKT/mTOR pathway under physiological conditions. Studies have shown that in ectopic endometrial tissues of AM, the expression level of PTEN is decreased, while the expression of the PI3K/AKT signaling pathway is increased (Xu et al., 2018; Zhang et al., 2022). When PTEN becomes dysfunctional or undergoes mutations, the PI3K/AKT/mTOR signaling pathway is activated. Inhibiting this pathway may represent an essential strategy for combating tumors (Yehia et al., 2020).

MicroRNAs (miRNAs) are a class of small, endogenous, highly conserved non-coding RNAs that regulate the expression of approximately 30% of genes at the post-transcriptional level, and this post-transcriptional regulation is linked to several pivotal biological processes, including cell proliferation, metastasis, autophagy, and apoptosis (Diener et al., 2022; Li et al., 2018). Among these, miR-21 is one of the most extensively studied and is recognized as a cancer promoter across various malignancies, correlated with tumor growth, metastasis, and poor survival in patients (Cao et al., 2019; Jiang et al., 2023; Sahraei et al., 2019). However, research on the role of miR-21 in the pathogenesis of AM remains relatively scarce. Juárez-Barber et al. (2023) pointed out that the expression of miR-21 is increased in the eutopic endometrium of patients with AM, and this is associated with the pathogenesis of AM and implantation failure. In the previous studies of our research group, it was found that the upregulation of miR-21 results in diminished PTEN expression, which subsequently leads to abnormal activation of the PI3K/AKT/mTOR pathway, which is involved in aberrant autophagy in EESCs (Jia et al., 2025).

In Traditional Chinese Medicine (TCM), AM is classified under the categories of “dysmenorrhea” and “abdominal masses”. The SGD, which consists of *Radix Paeoniae Alba* (Baishao, White Peony Root) and *Glycyrrhiza uralensis Fisch* (Zhigancao, Licorice Root), is a famous classical prescription from the Treatise on Febrile Diseases. For thousands of years, it has been clinically used to treat spasm-related and pain-related disorders caused by Qi and blood insufficiency as well as tendon and vessel malnutrition, especially in gynecological diseases (Facheng et al., 2025). Zhou et al. (2023) demonstrated that SGD can inhibit the proliferation of the human gastric adenocarcinoma cell line through the PI3K/AKT signaling pathway. Preliminary studies conducted by our research group have shown that SGD inhibits the proliferation and migration of EESCs and promotes apoptosis (Kuang et al., 2023). Although the inhibitory effect of SGD on the proliferative behavior of EESCs has been observed, the underlying potential mechanism deserves further clarification. In the present study, we highlight further evidence demonstrating the proapoptotic and proautophagic effects of SGD on EESCs in the area of miRNAs.

**FIGURE 1:**
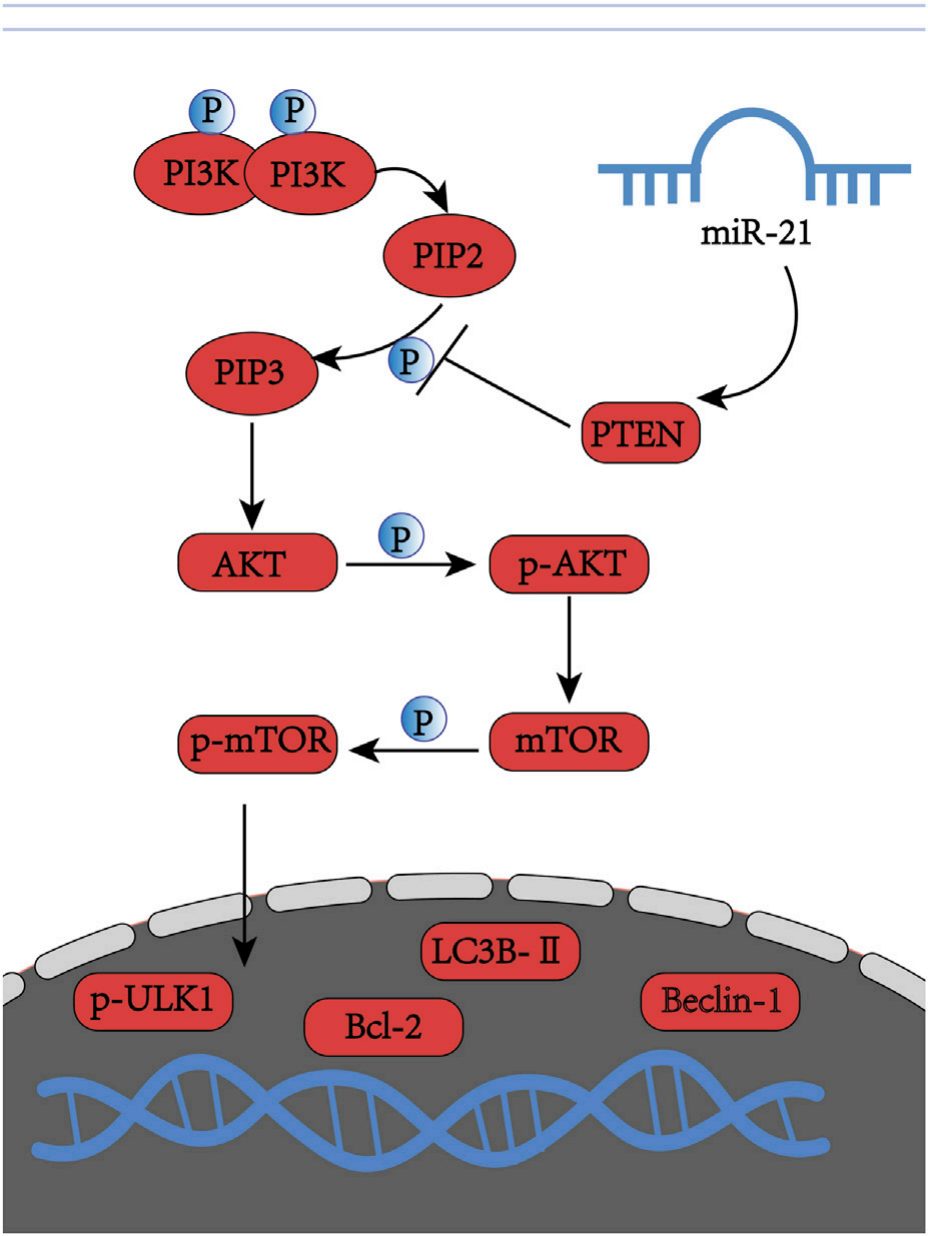
The regulatory cascade schematic.

Multiple studies have confirmed that PTEN is a target gene of miR-21 (Dong et al., 2017; Liu et al., 2019; Lu et al., 2015), and miR-21 promotes cell proliferation by sustaining the activation of the PTEN/PI3K/AKT pathway in cancer cells (Jinhua et al., 2022). Thus, a potential therapeutic strategy for interfering with the development and progression of AM involves inhibiting miR-21 expression to suppress the activation of PI3K/AKT/mTOR, thereby modulating the balance between proliferation and apoptosis. However, it remains unclear whether SGD regulates the activity of miR-21 and the PI3K/AKT/mTOR pathway in EESCs, nor is it known whether the effect of SGD on autophagy in EESCs is associated with the PI3K/AKT/mTOR signaling pathway regulated by miR-21. In the present study, we provide further evidence demonstrating the proapoptotic and proautophagic effects of SGD on EESCs in the field of miRNAs. Specifically, we clarified whether miR-21 is involved in SGD-mediated autophagy induction via the PI3K/AKT/mTOR signaling pathway. The regulatory cascade schematic is shown in Figure 1.

## 2 Materials and methods

### 2.1 Ethics approval and consent to participate

The project received approval from the Ethics Committee of Guangdong Provincial Hospital of Traditional Chinese Medicine (approval number: BF2020-123-01). The study was conducted in accordance with the 1964 Declaration of Helsinki and its subsequent amendments, as well as the Ethical Guidelines for Medical Research Involving Human Subjects. Written informed consent was obtained from all participants prior to their involvement in this study.

### 2.2 Specimen collection, and preservation

Four types of tissues, namely, AM ectopic endometrium (AM ectopic lesions), AM eutopic endometrium, normal myometrium, and normal endometrium, were collected for subsequent detection. The AM ectopic and eutopic endometrium were obtained from patients with AM who undergoing total hysterectomy between February 2023 and June 2023 at Guangdong Provincial Hospital of Traditional Chinese Medicine. At the same time, normal myometrium and normal endometrium were obtained from patients with Cervical Intraepithelial Neoplasia Grade 3 (CIN3) who undergoing total hysterectomy. All participants were premenopausal and had abstained from any estrogen/progesterone-based hormonal therapy for 3 months preceding tissue specimens collection. All tissue samples were authenticated by two experienced pathologists from the Department of Pathology at Guangdong Provincial Hospital of Traditional Chinese Medicine. Both participant groups were free from endometrial-related diseases and other gynecological conditions (such as uterine fibroids, endometrial cancer, cervical cancer). Tissue samples meeting these criteria were therefore subjected to subsequent analyses. Tissue specimens were collected aseptically in the operating room and subsequently frozen at −80 °C for storage. Additionally, during tissue sample collection, six cases of AM ectopic endometrium were selected for primary culture of EESCs. Successfully cultured EESCs were harvested and cryopreserved in liquid nitrogen tanks for subsequent applications.

### 2.3 Reagents and consumables

Dulbecco’s Modified Eagle’s Medium (DMEM, Servicebio, No. G4510); Fetal Bovine Serum (FBS, Gibco, No. 10099-141); Trypsin-Ethylene diamine tetra acetic acid (EDTA, 0.25%) Solution; Phenol Red (Gibco, No. 25200056); Collagenase Type I (0.1%, Sigma, No. C0130); SYBR Premix EX Taq II Kit (TaKaRa, No. RR820A); PrimeScript™RT Reagent Kit (TaKaRa, No. RR047A); 1X Phosphate-Buffered Saline (PBS, Biosharp, No. BL302A); Dimethyl Sulfoxide (DMSO, MP Biomedicals, No. 196055); Horseradish Peroxidase (HRP)-Conjugated Secondary Antibody (Bioworld, No. BS13278); Trizol Reagent (Ambion, No. 15596-026); PVDF Membrane (Millipore, IPVH00010); TBST; PMSF; TEMED; Tween-20 (Servicebio); Tris Base(BIOFROXX, 1115GR500); BCA Protein Quantification Kit (Gbs, No.G3522-3), and Bovine Serum Albumin Standard (Gbs No.G3018), among others.

### 2.4 Detection the mRNA expression of miR-21, AKT, PI3K, and mTOR in tissues by real-time quantitative reverse transcription polymerase chain reaction (RT-qPCR)

Total RNA extraction was conducted in accordance with the TRIzol user manual provided by Invitrogen. The purity and integrity of the extracted total RNA were confirmed following treatment with RNase-free DNase I (Promega). cDNA synthesis was performed using 4 μL of total RNA per sample. PCR reactions were carried out utilizing SYBRTM Premix Dimer Eraser (2×) at a volume of 10 μL, along with 0.6 μL each of 5′ and 3′ primers, and supplemented with 6.8 μL of PCR-grade distilled water. The reaction conditions commenced with an initial denaturation step at 95 °C for 3 min, followed by 40 cycles consisting of denaturation at 95 °C for 15 s and annealing/extension at 60 °C for 30 s. Primers specific to miR-21, AKT, PI3K, mTOR, as well as the reference gene beta-actin were synthesized by Sangon Biotech Company (China). A negative control reaction was established to ensure accuracy; subsequently, the relative expression levels of these genes within the sample tissues were calculated using the formula 2^−ΔΔCt^. The primer sequences utilized in this study are provided in Supplementary Table S1.

### 2.5 Detection the protein expression of PTEN/PI3K/AKT/mTOR signaling pathway in tissues by Western blotting (WB)

Tissues were homogenized in lysis solution before being centrifuged at 12,000 g. The supernatants were collected and the total proteins were extracted according to the P1250 kit instructions (APPLYGEN, Beijing, China). Concentrations of the proteins were determined using the BCA protein assay kit. Sulfate-polyacrylamide gel electrophoresis (SDS-PAGE) running buffer (5X) was added 4:1 to protein samples, which were kept in a boiling water bath for 10 min to fully denature. After being subjected to SDS-PAGE, proteins were then transferred onto a polyvinylidene fluoride membrane. The membranes were blocked at room temperature for 2 h in 5% skim milk powder. After incubation with primary antibodies (Table 1) overnight at 4 °C, membranes were washed for 10 min with PBST for 10 min. Incubation with secondary antibodies (1:40,000) took 2 h at room temperature, followed by 3 PBST washings for 10 min each time. Strips were detected with ECL chemiluminescent kits and analyzed with the ImageJ software.

**TABLE 1:** List of primary antibodies used for Western blot analysis.

Antibodies	Species	Molecular weight (kDa)	Batch no.	Manufacturer	Concentration
PTEN	Rabbit	54	Ab267787	Abcam	1:1,000
PI3K	Rabbit	123	Ab151549	Abcam	1:1,000
AKT	Rabbit	60	#4691	CST	1:1,000
p-AKT	Rabbit	60	#4060	CST	1:2,000
mTOR	Rabbit	250	Ab32028	Abcam	1:1,000
p-mTOR	Rabbit	289	Ab109268	Abcam	1:1,000
Bcl-2	Rabbit	26	Ab32124	Abcam	1:1,000
p-ULK1	Rabbit	112	Ab203207	Abcam	1:1,000
LC3B	Rabbit	15	Ab63817	Abcam	1:1,000
Beclin-1	Rabbit	60	#14717	CST	1:1,000
GAPDH	Rabbit	36	Ab181602	Abcam	1:10,000

### 2.6 Preparation of SGD-containing serum

#### 2.6.1 Preparation of SGD

The preparation of SGD follows the original proportions as detailed in Table 2. For animal gavage, SGD is formulated using 75 g of Radix Paeoniae Alba and 30 g of Glycyrrhiza Uralensis Fisch, amounting to a total of five doses. The botanical drugs were procured in small packages from the Guangdong Provincial Hospital of Traditional Chinese Medicine (No. 2111005, G042171). After the botanical drugs were crushed into powder and re-proportioned, the mixed drugs were boiled with 8 times the amount of distilled water, then simmered over low heat for 50 min. The decoction was concentrated to a fluid extract at 80 °C and made up to 1 mL containing 0.3 g of crude drugs to obtain the aqueous decoction of SGD.

**TABLE 2:** The compositions of SGD.

Latin name	Chinese name	Medicinal part	Daily adult dose (g)
*Radix Paeoniae Alba*	Baishao	Dried rhizomes	15 g
*Glycyrrhiza Uralensis Fisch*	Zhigancao		6 g

#### 2.6.2 Chemical analysis of SGD by UPLC-HRMS detection

SGD extracts were analyzed using a Vanquish UHPLC system (Thermo Scientific, Waltham, MA) equipped with a HSS-T3 column (100 × 2.1 mm, 1.8 μm particles size; Waters) at a column compartment termperature of 35 °C. Mobile phase A is H2O+0.1%formic acid and mobile phase B is acetonitrile+0.1%formic acid (LC-MS grade solvents, Fisher chemical). Samples were separated with a flow rate of 0.3 mL/min using the following gradient: 1 min isocratic at 5% B, up to 98 B in 16 min, back to 5% B in 0.5 min and then 2.5 min isocratic at 5% B.

Q-Exactive HFX mass spectrometer (Thermo Fisher Scientific, Bremen, Germany) was coupled to the UHPLC system. Mass spectra were acquired in both electrospray ionization (ESI) positive and negative modes using data-dependent acquisition (DDA) mode with a mass range of m/z 90-1,300. The MS/MS spectra were obtained from the top 10 most intense MS1 ions. The stepped normalized high-energy dissociation (HCD) collision energies (CEs) of 20, 40, and 60 units were used. Capillary temperature is 320 °C and probe heater temperature is 350 °C.

#### 2.6.3 Animal grouping and preparation of SGD-containing serum

A total of 10 SPF-grade female SD rats, weighing 200 ± 20 g were utilized (Provided by Guangdong Experimental Animal Center, Certificate of Animal Conformity: N0.4400720013883, Animal ethics license number: SCXK-0002). The dosage of drug administration for rats was calculated as five times the equivalent dose conversion factor of standard animals in the Pharmacological Experiment Methodology. In the previous experiments of the research group, the SGD was divided into low (1.15 × 10^3^ mg/(kg·d)), medium (2.31 × 10^3^ mg/(kg·d)), and high (4.62 × 10^3^ mg/(kg·d)) doses to prepare the drug-containing serum. The results showed that after 48 h of culture, compared with the low and medium-dose SGD-containing serum groups, the EESCs in the high-dose group had the lowest cell migration rate and the highest cell apoptosis rate. Therefore, high-dose SGD-containing serum was used for the subsequent experiments in this study (Kuang et al., 2023).

After 3 days of adaptive feeding, rats were randomly divided into the blank serum group (n = 5) and the SGD-containing serum group (n = 5). The preparation of drug serum adheres to our previous work (Guan Y.-G. et al., 2014). After the concentration of SGD was set, the drug group was given 2 mL of SGD solution by gavage, while the blank serum group was given the same volume of normal saline. Continuous intragastric administration for 5 days, twice a day in the morning and evening. On the 5th day of administration, the rats were fasted but allowed to drink water. All rats were anesthetized 1 h after the last gavage, and blood samples were collected from the abdominal aorta. The blood samples were allowed to clot at 25 °C and centrifuged at 3,000 rpm for 10 min. Reserve the supernatant for later use. The serum was subsequently inactivated in a water bath at 56 °C for 30 min. After being filtered and sterilized with a 0.22 μm filter membrane, the serum was stored at −80 °C for subsequent cell experiments.

### 2.7 Culture, passaging, and identification of EESCs and grouping

AM ectopic endometrium was collected under sterile conditions during surgical procedures and preserved in a pre-cooled PBS solution, which was subsequently transported to the laboratory for primary cell culture. According to post-surgical pathological diagnosis, specimens that did not meet the inclusion criteria were excluded. Following collection, tissues were washed 3 times with PBS, cut into 1 mm^3^ pieces with scissors, and transferred into 50 mL centrifuge tubes. After being treated with approximately twice the volume of type I collagenase solution for 4–5 h, an equal volume of DMEM medium (containing 10% FBS) was added to terminate the digestion process.

Supernatants were collected by filtering the cell suspension through a mesh and subsequently centrifuging at 1,000 rpm for 8 min. DMEM medium containing 10% fetal bovine serum (FBS), 100 U/mL penicillin, and 0.1 mg/mL streptomycin was used for EESCs cultures. The EESCs were incubated in a 37 °C with 5% CO_2_ incubator. The culture medium should be replaced after 48 h, and then changed every 2–3 days thereafter. The growth of the cells should be observed. When cell confluence in the culture dish exceeded 90%, subculturing was performed. The passaged cells were harvested, transferred into a coolcell controlled-rate container, and cryopreserved in a −80 °C freezer for subsequent experimental use. EESCs were stained and identified using immunocytochemistry (ICC) during the first passage of primary cells. The characteristic vimentin of interstitial cells was employed to identify EESCs.

### 2.8 miR-21 transfection of EESCs

The miR-21 mimic is a synthetic analog that induces overexpression of miR-21, while the miR-21 inhibitor suppresses endogenous miR-21 expression. Following resuscitation of cryopreserved cells, viable cells exhibiting optimal growth activity were seeded into 6-well cell culture plates at a density of 5 × 104 cells per well and incubated overnight at 37 °C with 5% CO_2_ incubator. The cells were divided into five experimental groups: EESCs group (control), EESCs + mimics negative control (NC) group, EESCs + miR-21 mimics group, EESCs + inhibitor NC group, and EESCs + miR-21 inhibitor group. (Hereinafter abbreviated as: control group; mimics NC group; miR-21 mimics group; inhibitor NC group; miR-21 inhibitor group).

For each transfection sample, the following preparation protocol was strictly implemented: 0.5 μL of siRNA (20 μM) was diluted in 10 μL serum-free Opti-MEM, gently mixed by pipetting, and incubated at room temperature for 5 min. Concurrently, 0.25 μL of thoroughly vortexed Lipofectamine™ 2000 was diluted in 100 μL Opti-MEM and incubated at room temperature for 5 min to allow reagent equilibration. The two diluted solutions (total volume: 200 μL) were combined and incubated at room temperature for 20 min. Subsequently, 20 μL of the complex mixture was aliquoted into each culture well. Plates were gently swirled and transferred to a 37 °C with 5% CO_2_ incubator. After 6 h, the transfection mixture was aspirated and replaced with complete growth medium. Cells were cultured for an additional 48 h under standard conditions.

### 2.9 TEM was employed to examine the ultrastructural features of EESCs following transfection

Following 48 h post-transfection, the experimental workflow proceeded as follows: Cells from each group were trypsinized, harvested, and resuspended in an equal volume of 2.5% glutaraldehyde fixative. After centrifugation at 2,000 rpm for 10 min, the supernatant was discarded. The pellet was reconstituted in 2.5% glutaraldehyde fixative containing 10% FBS, gently dispersed via pipetting, and recentrifuged under identical conditions. The supernatant was removed, and cells were progressively fixed in a hybrid solution containing 2.5% glutaraldehyde, 2% paraformaldehyde, and 0.1% phosphate buffer for 30 min. Post-fixation, samples underwent three PBS washes (3 min each). Secondary fixation was performed using 1 g/L osmium tetroxide for 30 min, followed by another PBS washing cycle. Gradual ethanol dehydration preceded embedding in resin, which was polymerized at 60 °C for 72 h. Ultrathin sections were prepared, dual-stained with uranyl acetate and lead citrate, and imaged under a TEM.

### 2.10 Detection the mRNA expression of miR-21 and PTEN in EESCs by RT-qPCR

After resuscitation of the cryopreserved cells, EESCs exhibiting optimal growth status were cultured for subsequent experiments. EESCs were transfected with miR-21 inhibitor or miR-21 mimic for 48 h, followed by culture in SGD-containing serum, respectively. The EESCs was divided into four groups: blank serum group (DMEM medium containing 10% FBS), SGD-containing serum group (DMEM medium containing 10% SGD-containing serum), SGD + miR-21 mimics Group, and SGD + miR-21 inhibitor Group.

The Trizol method was used to extract RNA and reverse transcribe it into cDNA. The reaction conditions were 50 °C for 15 min, 85 °C for 5 s, and 4 °C for 10 min cDNA was synthesized with 4 μL of total RNA per sample. PCR reactions were performed with SYBRTM Premix Dimer Eraser (2×) 10 μL, 0.4 μL of 5′and 3′primers, and 5.2 μL PCR-grade distilled water. The reaction conditions were started at 95 °C for 10 min for initial denaturation and then followed by 40 cycles of 10 s at 95 °C and 60 s at 60 °C. Primer design was performed as described in 2.4. The Primer sequences used in the present study are listed in Supplementary Table S2.

### 2.11 Detection of apoptosis in EESCs by flow cytometry

Flow cytometry (FCM) was used to detect the apoptosis rate of EESCs in each group, with the grouping method consistent with that described in Section “2.10”. Add corresponding serum according to the above grouping and incubate in a 37 °C with 5% CO_2_ incubator for 48 h. The cells were washed twice with PBS and then digested and collected from each group using trypsin. After centrifuging at 1,200 r/min for 5 min, the supernatant was discarded, and 500 μL of Binding Buffer was added to resuspend the cells. Subsequently, 5 μL of AnnexinV-APC was added, followed by mixing and then the addition of 5 μL of 7-AAD. After incubating in the dark at room temperature for 5–15 min, the cells were analyzed on a flow cytometer to calculate the apoptosis rate.

### 2.12 Detection of migration distance in EESCs by cell scratch test

Cell scratch test was employed to detect the migration level of EESCs in each group. Forty-eight hours after cell transfection, cells in each group were separately digested with trypsin and collected. Using a marker pen and a ruler, evenly spaced horizontal lines were drawn on the back of 6-well plates. Subsequently, the cells were seeded into 6-well plates at a density of 1 × 10^6^ cells/well and cultured overnight in a cell incubator at 37 °C with 5% CO_2_. Once the cell density exceeded 90%, scratches were made with a pipette tip along the ruler, perpendicular to the horizontal lines on the back. The cells were washed 3 times with PBS to remove detached cells, and then culture media containing corresponding sera were added according to the grouping described in Section “2.10”. For each group, samples were photographed under a ×40 objective lens at 0 h and 24 h, respectively.

### 2.13 Detection of proliferation rate in EESCs by MTT

3-(4,5-Dimethylthiazol-2-yl)-2,5-diphenyltetrazolium bromide assay (MTT) was employed to detect the proliferation rate of EESCs in each group. Forty-eight hours after cell transfection, the medium was replaced with culture media containing different sera. Ten microliters of MTT solution was added to each well, and the plates were incubated in a 37 °C, 5% CO_2_ incubator for 2 h. After incubation, the medium in each well was aspirated, and 150 μL of DMSO was added to each well. The plates were shaken for 10 min to fully dissolve the crystals. Finally, the absorbance of each well was measured at a wavelength of 570 nm (A_570_) using a microplate reader. The cell growth and proliferation rate was calculated according to the following formula:

$$\text{Cell proliferation rate }\left(\%\right)=[\left(A_{\text{experimental}}-A_{\text{black}}\right)/\left(A_{\text{control}}-A_{\text{black}}\right)]\times 100\%$$

### 2.14 Detection the protein expression in EESCs by WB

In this part, the EESCs were divided into three groups: the control group (DMEM medium), the blank serum group (DMEM medium containing 10% FBS), and the SGD-containing serum group (DMEM medium containing 10% SGD serum). The EESCs were thoroughly lysed with prepared RIPA lysis buffer, and after lysis, the protein was transferred to a 1.5 mL EP tube and centrifuged at 12,000 rpm for 5 min at 4 °C. The concentration of total proteins was measured according to the instructions of the BCA Protein Quantification Kit. The total protein was subjected to SDS-PAGE at 80 v for the stacking gel and 120v for the resolving gel for 90 min, followed by a semi-dry transfer at 1.5A for 420 s. After the membrane was blocked with 5% skim milk-TBST for 2 h at room temperature, the phosphorylated protein was blocked with 1% BSA. It was incubated with primary antibodies (Table 1) and kept on a rocker in darkness overnight, at 4 °C. On the second day, corresponding secondary antibodies at 1:10,000 dilution were added for incubation for 2 h at 37 °C. After washing away the secondary antibody, perform color development exposure, air dry and scan the film, and analyze the grayscale value of the film using IPP.

### 2.15 Statistics

SPSS26.0 software was used for statistical analysis of data. First, Levene’s homogeneity of variance test was used to analyze the continuous data. If the data satisfied assumptions of normality and homogeneity of variance, results were presented as the mean ± standard deviation, and the overall difference was compared by the analysis of variance method. When the differences between populations were statistically significant, the least significant difference (LSD) test was used for pairwise comparisons. If the data did not meet the homogeneity of variance, results were presented as the median (interquartile range), and the Wilcoxon score was used, followed by the Kruskal-Wallis test. *P < 0.05* was considered as a statistically significant difference.

## 3 Results

### 3.1 Identification of chemical compositions in SGD

**FIGURE 2:**
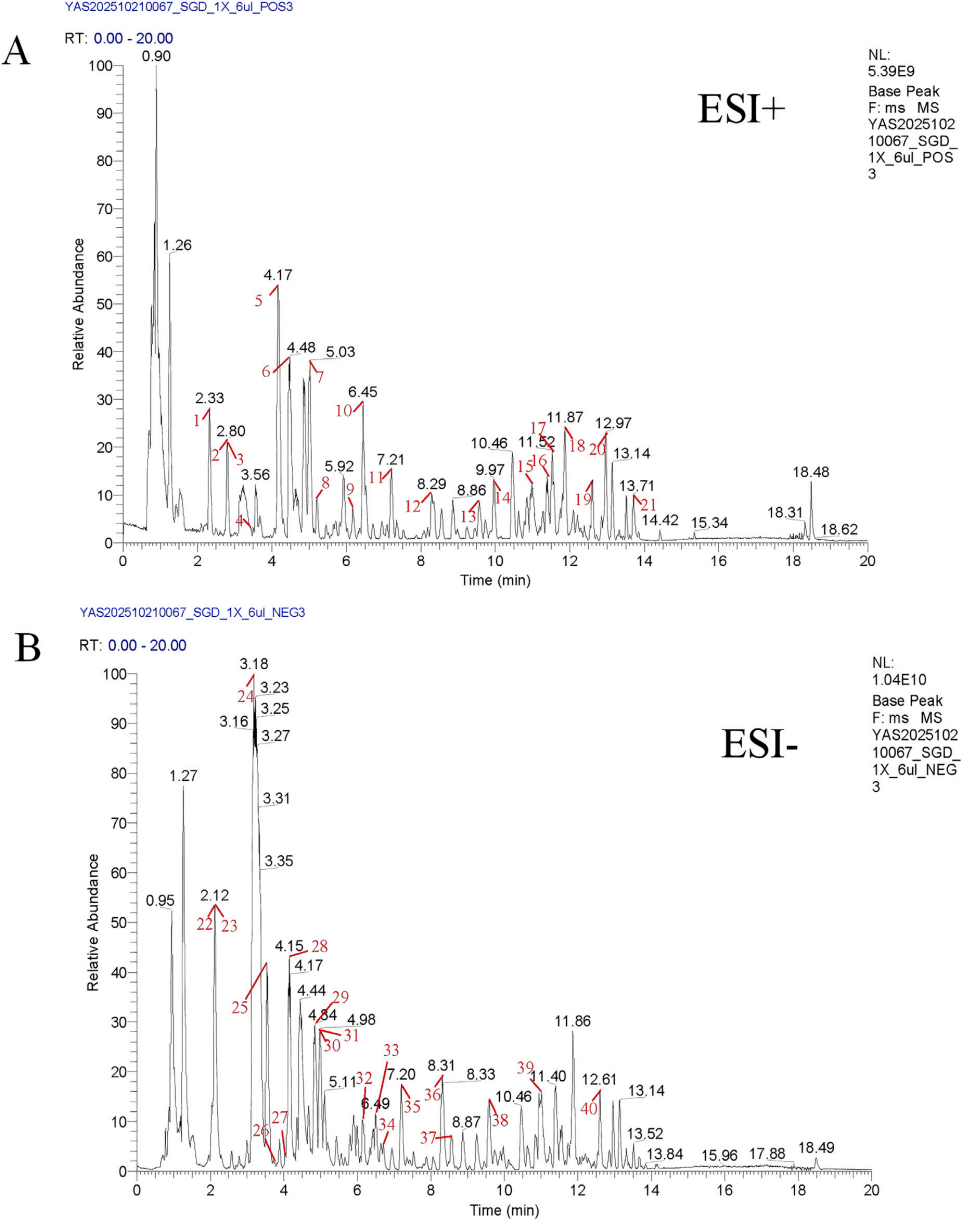
UPLC-HRMS identification results of the main chemical components of SGD. **(A)** BPC of SGD in positive ion mode; **(B)** BPC of SGD in negative ion mode.

In the positive and negative ion base peak chromatograms (BPC) of SGD, the chromatographic peaks with higher abundance were subjected to peak shape confirmation and MS/MS spectra verification. The peaks in the positive and negative ion chromatograms were then sequentially labeled with numerical indices, as shown in Figure 2. In this project, a total of 40 chromatographic peaks identified from traditional Chinese medicine were labeled, and the corresponding compound information is provided in the Supplementary Table S3.

### 3.2 Differential expression of miR-21 and PI3K/AKT/mTOR pathway mRNAs in tissues

**FIGURE 3:**
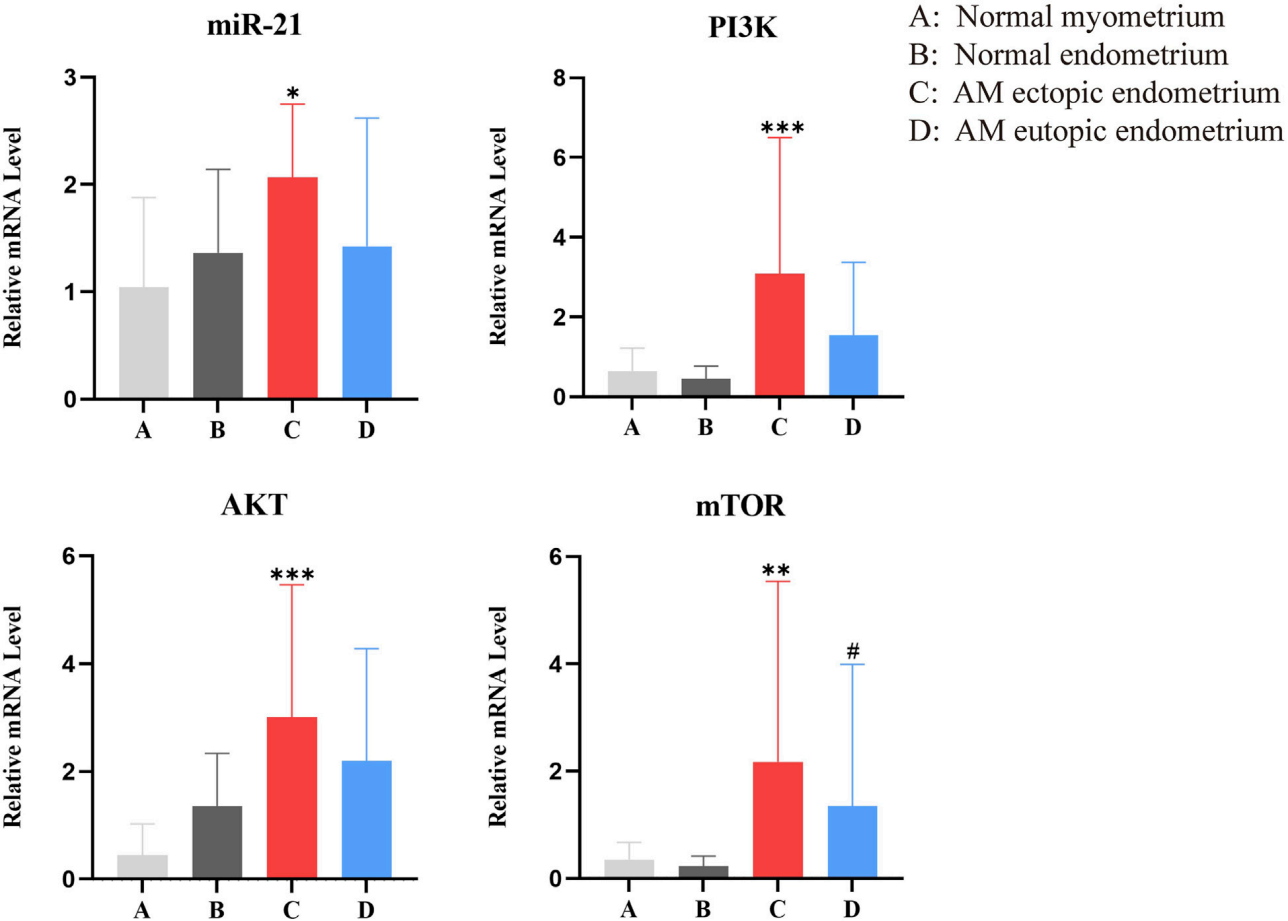
The expression of miR-21, PI3K, AKT, and mTOR mRNA in four tissues. *, compared with normal myometrium, *P < 0.05; **P < 0.01; ***P < 0.001; #, compared with normal endometrium, #*P* < 0.05.

A total of 28 samples of AM ectopic endometrium and normal myometrium tissues, as well as 12 samples of AM eutopic endometrium and normal endometrium tissues, were collected. The expressions of miR-21, PI3K, AKT, and mTOR mRNA were significantly elevated in the AM ectopic endometrium when compared to normal myometrium (*P* < 0.05), as depicted in Figure 3. However, compared to the normal endometrium, there was no statistically significant difference in the expression of miR-21, PI3K, and AKT mRNA observed in the AM eutopic endometrium (*P* > 0.05), except for mTOR mRNA (*P* < 0.05).

### 3.3 Differential expression of PTEN/PI3K/AKT/mTOR signaling pathway proteins in tissues

**FIGURE 4:**
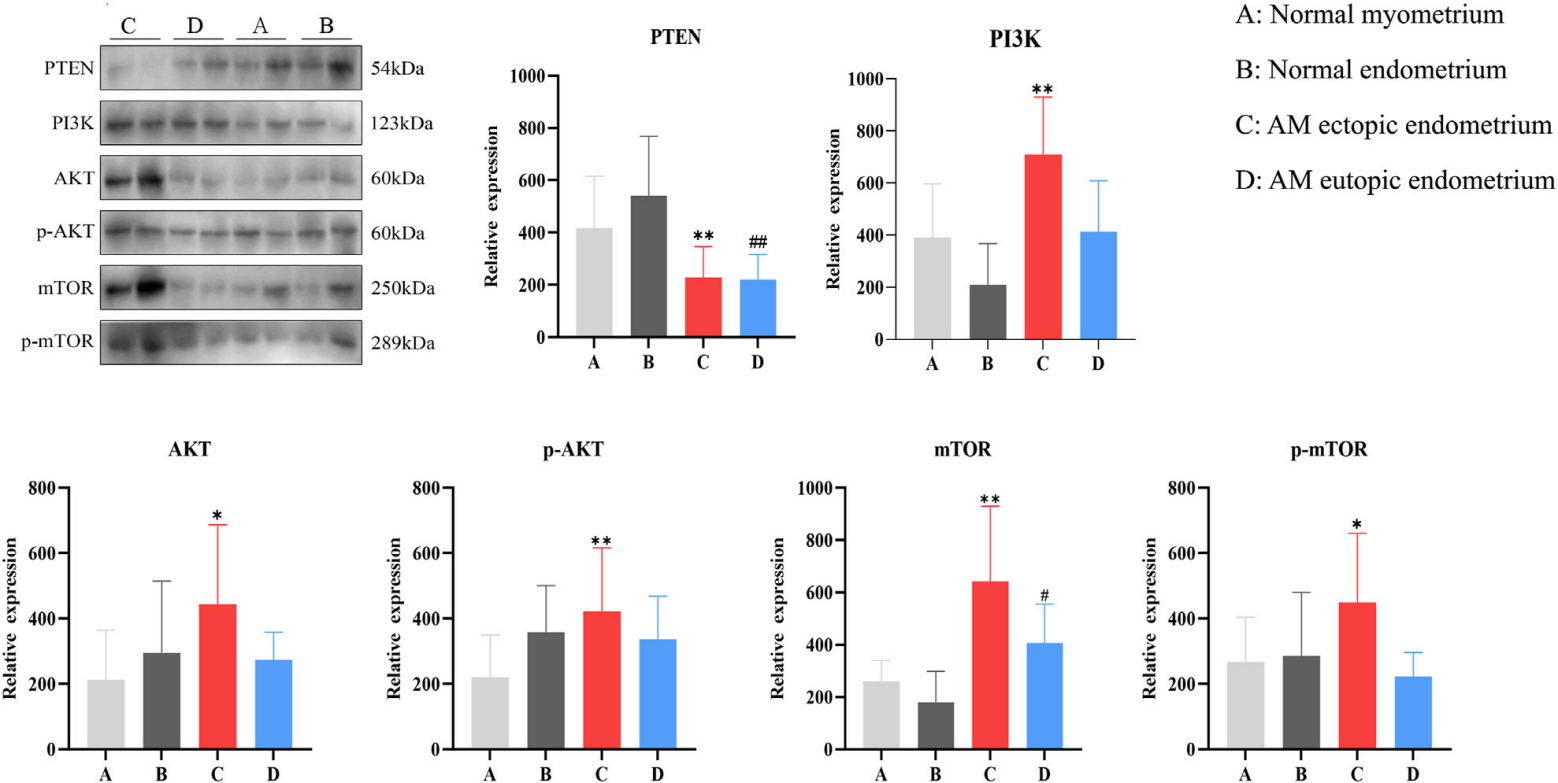
Expression of PI3K/AKT/mTOR pathway proteins in four tissues samples. *, compared with normal myometrium, *P < 0.05, **P < 0.01; #, compared with normal endometrium, #*P* < 0.05, ##*P* < 0.01.

The expression of proteins associated with the PTEN/PI3K/AKT/mTOR signaling pathway was assessed across four types of tissue samples, as illustrated in Figure 4. Notably, when compared to normal myometrium, the expression of PI3K, AKT, p-AKT, mTOR, and p-mTOR proteins was significantly elevated in AM ectopic endometrium (*P* < 0.05), whereas the PTEN protein was found to be decreased (*P* < 0.01). Additionally, the expression of PTEN was lower (*P* < 0.05), while mTOR expression levels were higher (*P* < 0.05) in the AM eutopic endometrium compared to those in normal endometrium. These results suggest that the activation of the PI3K/AKT/mTOR signaling pathway in AM ectopic endometrium may be associated with a reduced expression of PTEN.

### 3.4 Inhibition of autophagy in AM ectopic endometrium

**FIGURE 5:**
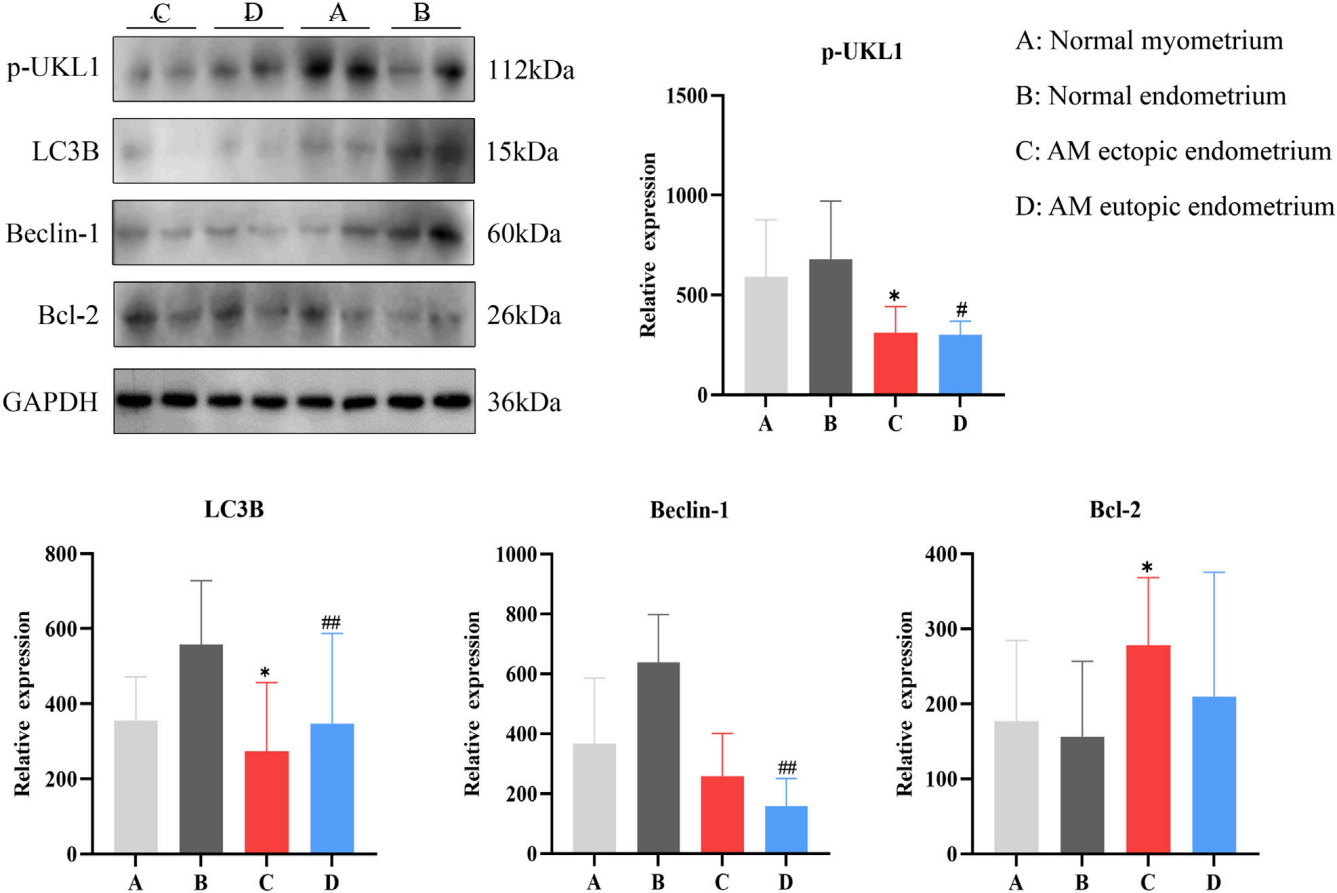
Expression of autophagy-related and apoptosis-related proteins in tissue samples. *, compared with normal myometrium, *P < 0.05; #, compared with normal endometrium, #*P* < 0.05, ##*P* < 0.01.

The expression levels of autophagy-related proteins in four types of tissue samples were illustrated in Figure 5. Notably, the expression of the autophagy markers Light chain 3B (LC3B) and p-UKL1 activating kinase 1 was significantly reduced (*P <* 0.05), while the anti-apoptotic protein B-cell lymphoma-2 (Bcl-2) exhibited a significant increase (*P <* 0.05) in AM ectopic endometrium compared to normal myometrium. When comparing AM eutopic endometrium to normal endometrium, there was a marked decrease in the expression of p-UKL1, LC3B, and Beclin 1 protein (Beclin-1) (*P <* 0.05, *P <* 0.01); however, the increase observed in Bcl-2 expression did not reach statistical significance. These findings suggest that autophagy is inhibited within AM ectopic lesions.

### 3.5 Culture and identification of EESCs

**FIGURE 6:**
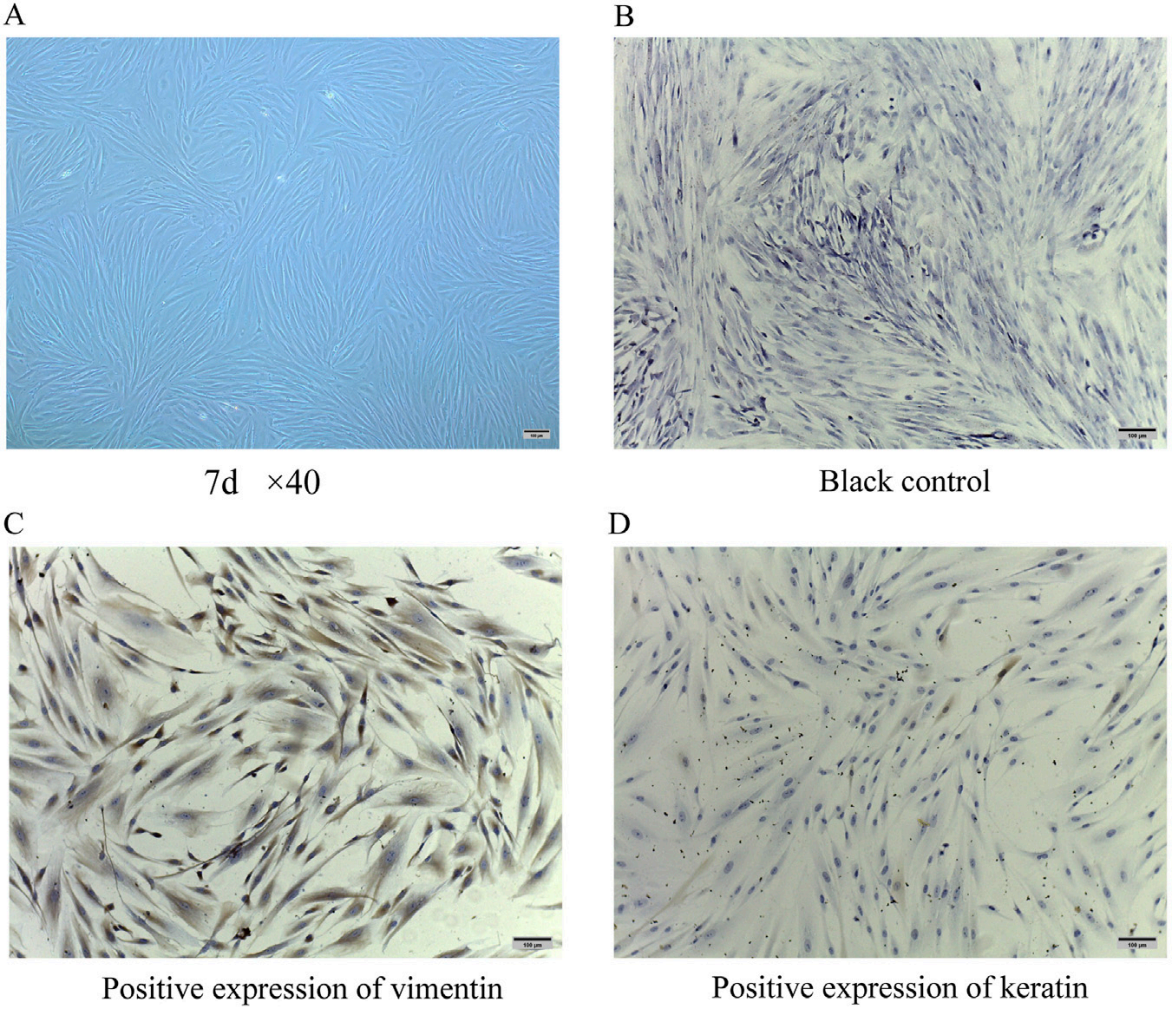
Culture and identification of EESCs. **(A)** Primary cell culture (inverted microscope ×40), the EESCs were cultured for 7 days. **(B–D)** Identification of EESCs (inverted microscope ×200).

Four cases of EESCs were successfully cultured, and immunocytochemistry was performed with the cultured cells of each case, as shown in Figure 6. After 48–72 h of primary cell extraction, some EESCs were observed to adhere and grow under microscopic examination. Throughout the culturing process, EESCs exhibited characteristic spindle-shaped morphology, arranged in parallel formations, and demonstrated robust vitality. Following cryopreservation and subsequent resuscitation, the cellular activity remained stable, with morphology closely resembling that of the primary cells. Immunocytochemical analysis revealed that the vimentin—a marker protein for interstitial cells—showed positive expression, while keratin—a marker for epithelial cells—showed negative expression. These results suggested that both the viability and purity of the EESCs are high, indicating their utility for further experimental studies.

### 3.6 miR-21 inhibits apoptosis of EESCs under TEM

**FIGURE 7:**
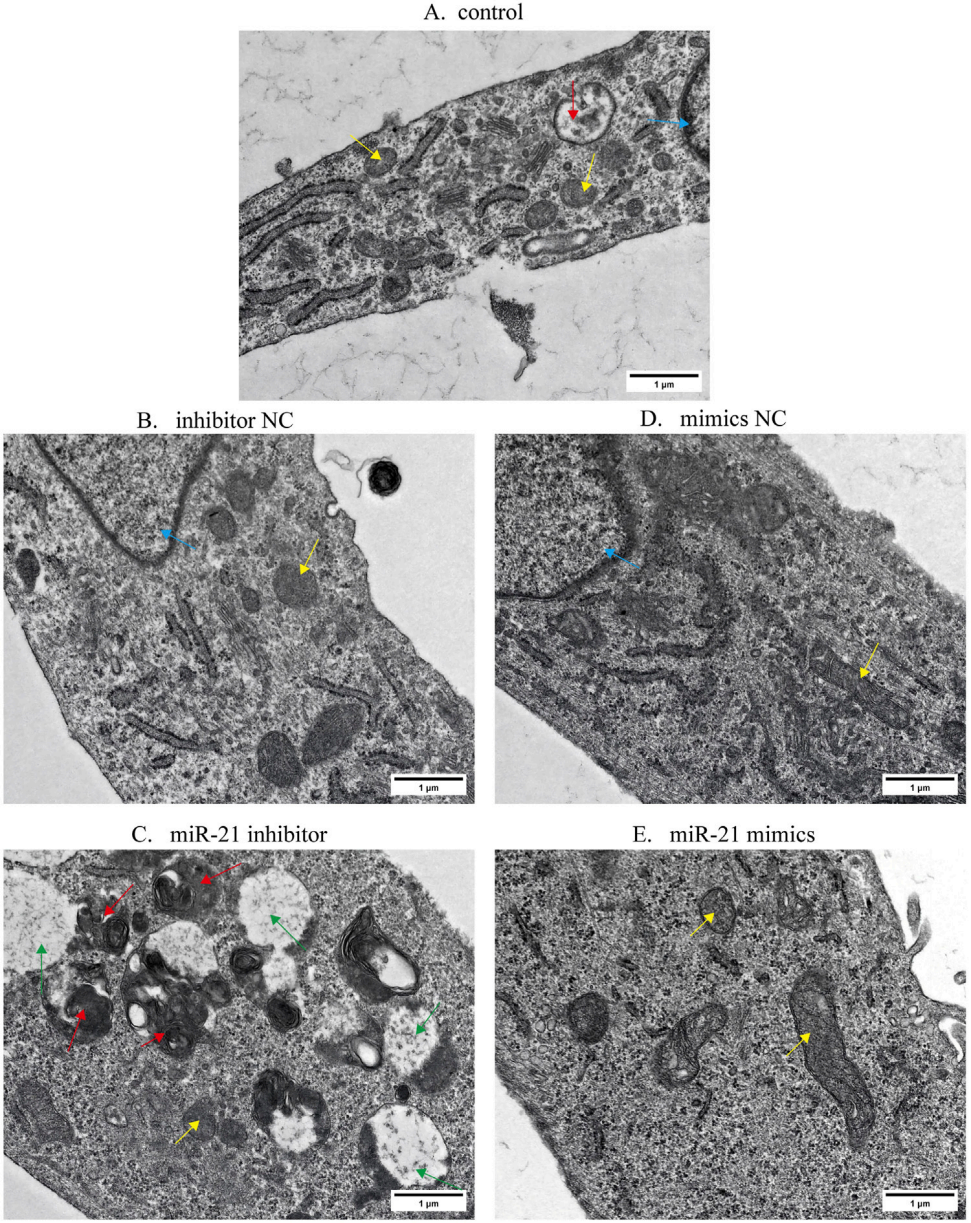
The ultrastructural alterations of EESCs in each group observed under TEM. Cellular structures are differentially labeled: apoptotic bodies (red arrows), normal mitochondria (yellow arrows), abnormally vacuolated mitochondria (green arrows), and the nucleus (blue arrows). **(A)** control. **(B)** inhibtor NC. **(C)** miR-21 inhibitor. **(D)** mimics NC. **(E)** miR-21 mimics.

After the EESCs were transfected for the same duration and then observed under a microscope, the number of EESCs in the miR-21-mimic group was significantly higher compared to the other three groups. Compared to the control group, the expression levels of miR-21 in the miR-21 mimic group were significantly elevated (*P* < 0.001), whereas those in the miR-21 inhibitor group were markedly reduced (*P* < 0.001). As illustrated in Figure 7, following transfection with miR-21 mimic, the ultrastructural characteristics of EESCs were analyzed via TEM.

In the control group, electron microscopy revealed regularly shaped nuclei with prominent nucleoli, abundant mitochondria displaying normal morphology in the cytoplasm, rare apoptotic bodies, and well-organized endoplasmic reticulum with intact structures. In contrast, the miR-21 inhibitor group exhibited increased cytoplasmic density with disorganized contents, containing numerous apoptotic bodies. Aggregation of ribosomes and mitochondria was observed, accompanied by reduced numbers of normal mitochondria showing irregular shapes and vacuolar degeneration. The endoplasmic reticulum appeared mildly dilated. Conversely, the miR-21 mimics group demonstrated abundant cytoplasmic components with numerous plump mitochondria maintaining normal architecture, absence of apoptotic bodies, and non-dilated endoplasmic reticulum structures.

### 3.7 Effects of SGD on miR-21 and PTEN mRNA in EESCs

**FIGURE 8:**
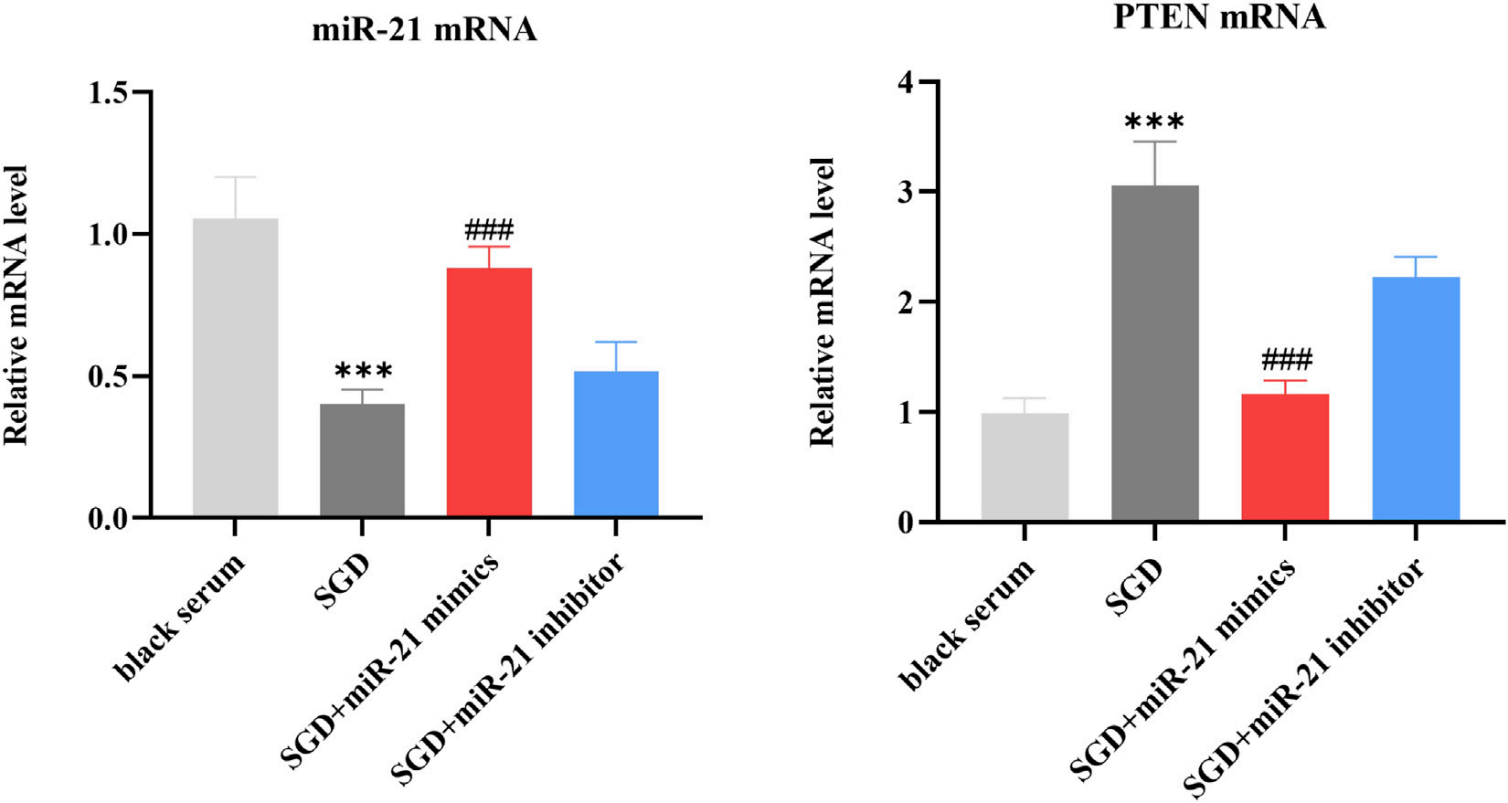
Effects of SGD on miR-21 and PTEN mRNA in EESCs. *, compared with black serum group, ***P < 0.001; #, compared with SGD group, ###*P* < 0.001.

The expression of miR-21 and PTEN mRNA was evaluated across different groups, as shown in Figure 8. Compared to the blank serum group, we observed a significant decrease in miR-21 mRNA levels, while there was an increase in PTEN mRNA levels after intervention with SGD-containing serum (*P <* 0.001; *P <* 0.001). When compared to the SGD-containing serum group, the expression of miR-21 mRNA was significantly increased in the SGD + miR-21 mimics group while PTEN mRNA was decreased. This result suggests that overexpressing miR-21 restores miR-21 levels after SGD treatment, thereby reversing the upregulation of PTEN expression.

### 3.8 The effect of SGD-mediated miR-21 downregulation on EESCs migration

**FIGURE 9:**
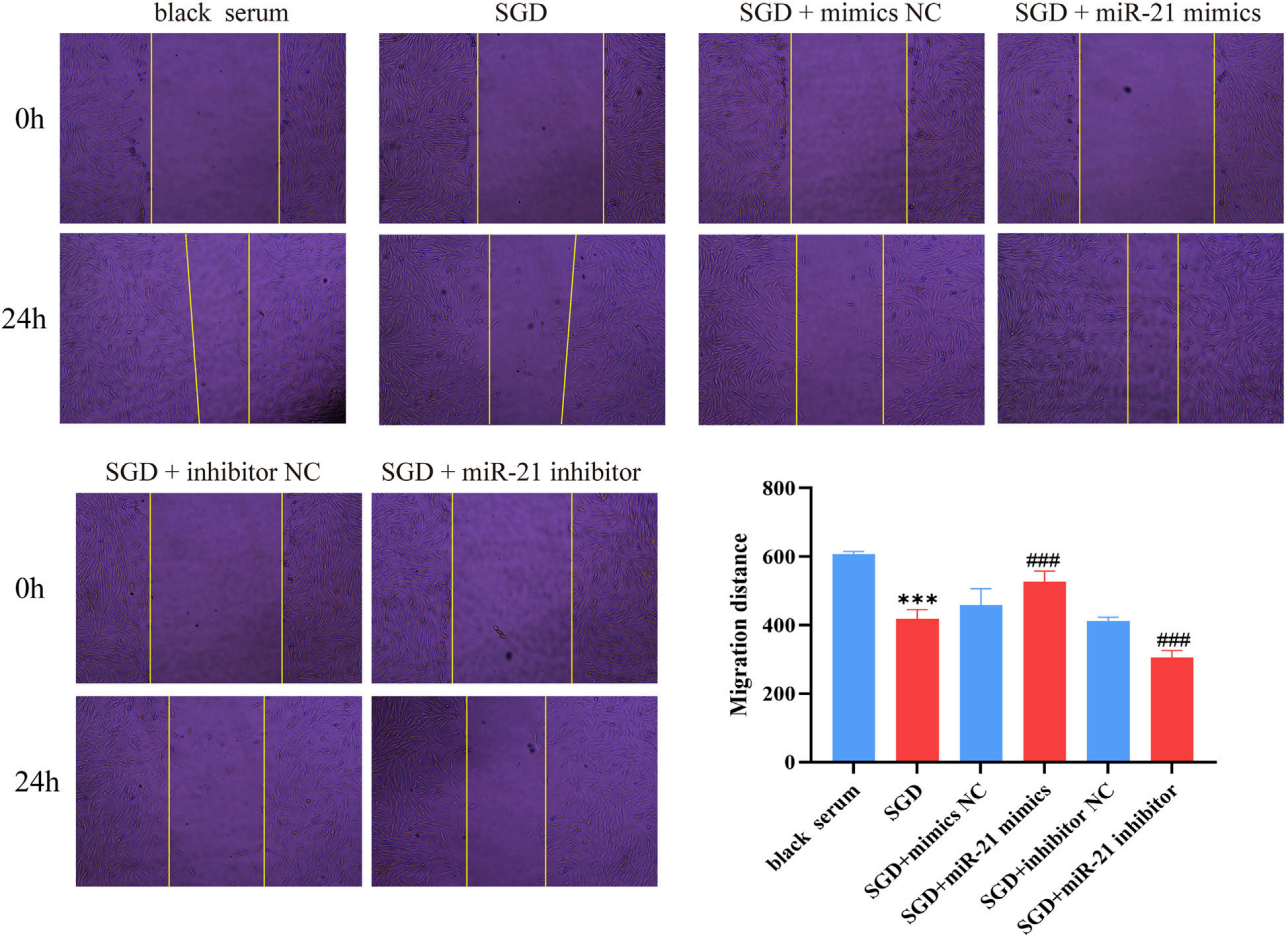
Cell scratch experimental results. *, compared with black serum group, *P* < 0.001; #, compared with SGD group, ###*P* < 0.001.

Next, we investigated the effect of SGD-mediated miR-21 downregulation on EESCs migration, with the results shown in Figure 9. Compared with the blank serum group, the migration distance of EESCs in the SGD-containing serum group (*P <* 0.001) was significantly reduced. In comparison to the SGD-containing serum group, the migration distance of EESCs in the SGD + miR-21 mimics group was significantly increased, while that in the SGD + miR-21 inhibitor group was significantly decreased. These results indicate that miR-21 mimics can reverse the SGD-induced reduction in EESCs migration distance, further confirming that SGD reduces EESCs migration by inhibiting miR-21.

### 3.9 The effect of SGD-mediated miR-21 downregulation on EESCs proliferation and apoptosis

**FIGURE 10:**
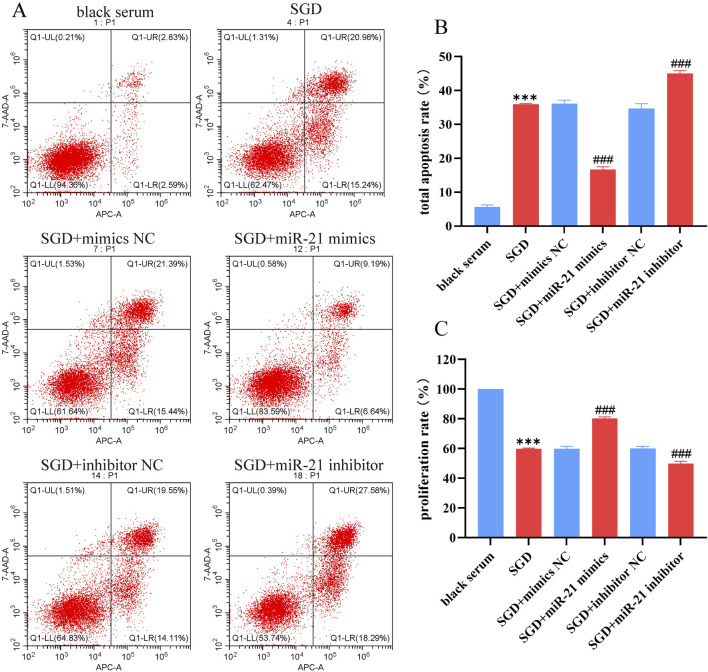
Effect of SGD-mediated miR-21 on the proliferation and apoptosis of EESCs. **(A)** Flow cytometry test chart of each group; **(B)** Histogram of total apoptosis rate in each group. **(C)** Histogram of proliferation rate in each group. *, compared with black serum group, *P* < 0.001; #, compared with SGD group, ###*P* < 0.001.

The effect of SGD-mediated miR-21 downregulation on EESCs apoptosis and proliferation was investigated simultaneously in this study, as shown in Figure 10. The results of flow cytometry for detecting cell apoptosis showed that compared with the blank serum group, SGD-containing serum significantly increased the apoptosis rate of EESCs; compared with the SGD-containing serum group, the apoptosis rate of EESCs in the SGD + miR-21 mimics group was significantly decreased, while that in the SGD + miR-21 inhibitor group was significantly increased.

The results of determining the relative proliferation rate of EESCs using MTT assay showed that compared with the blank serum group, the proliferation rate of EESCs in the SGD-containing serum group was significantly decreased; compared with the SGD-containing serum group, the proliferation rate of EESCs in the SGD + miR-21 mimics group was significantly increased, while that in the SGD + miR-21 inhibitor group was significantly decreased.These results further show that SGD inhibits the proliferation of EESCs and promotes their apoptosis by reducing the expression of miR-21.

### 3.10 Effects of SGD on PTEN/PI3K/AKT/mTOR pathway proteins in EESCs

**FIGURE 11:**
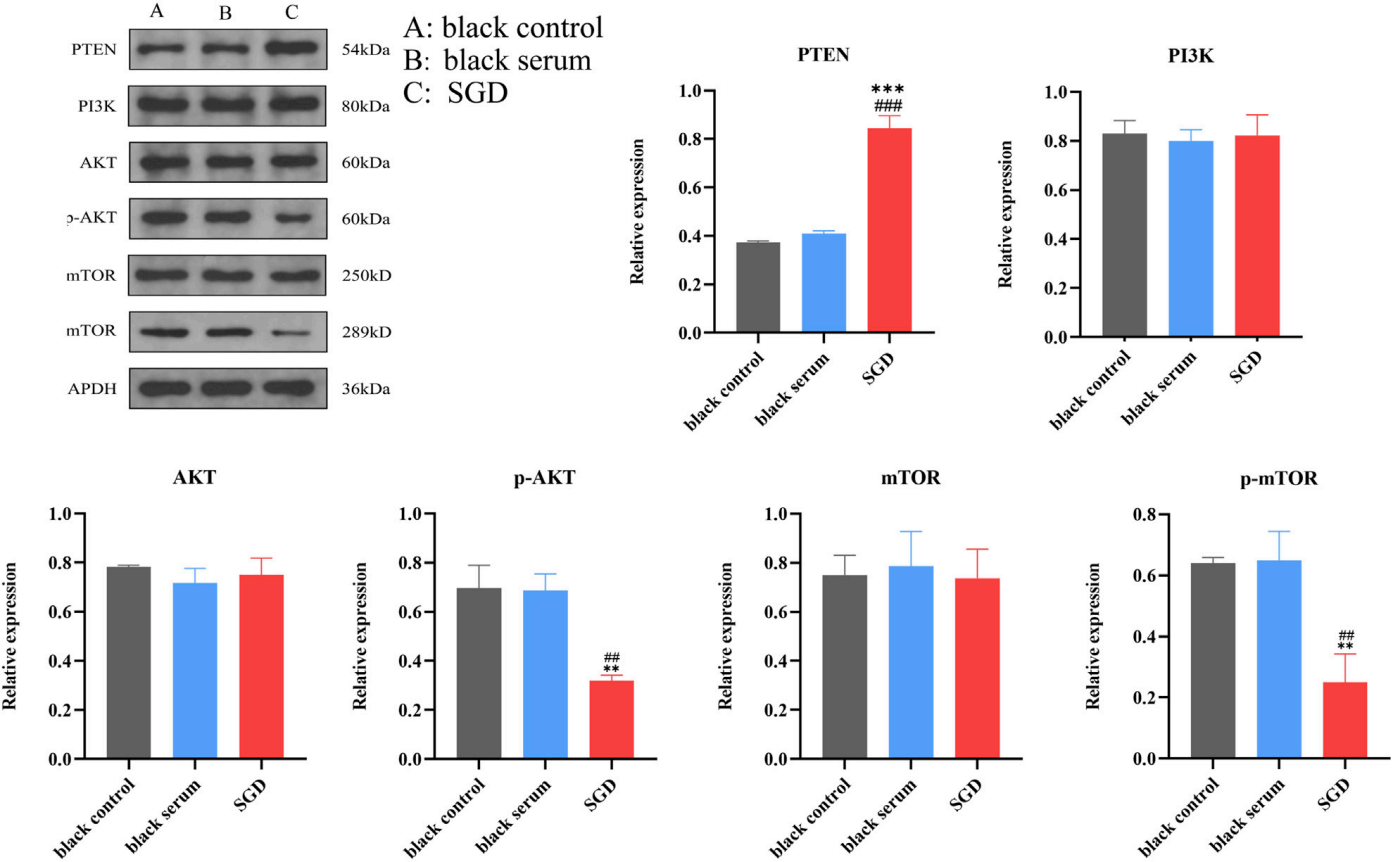
Effects of SGD on PTEN/PI3K/AKT/mTOR pathway proteins in EESCs. *Compared with the control group. *P* < 0.05, **P < 0.01, ***P < 0.001; #, compared with blank serum group, ##*P* < 0.01,###*P* < 0.001.

The PTEN/PI3K/AKT/mTOR pathway proteins in three groups of EESCs were detected, as illustrated in Figure 11. Compared to both the blank control and blank serum groups, SGD-containing serum resulted in a significant decrease in the expression of p-AKT and p-mTOR proteins (*P* < 0.01), while PTEN protein expression was markedly elevated (*P* < 0.001). However, no statistically significant difference was observed in PI3K, AKT, and mTOR expression levels (*P* > 0.05) in three groups.

### 3.11 SGD promotes autophagy and apoptosis in EESCs

**FIGURE 12:**
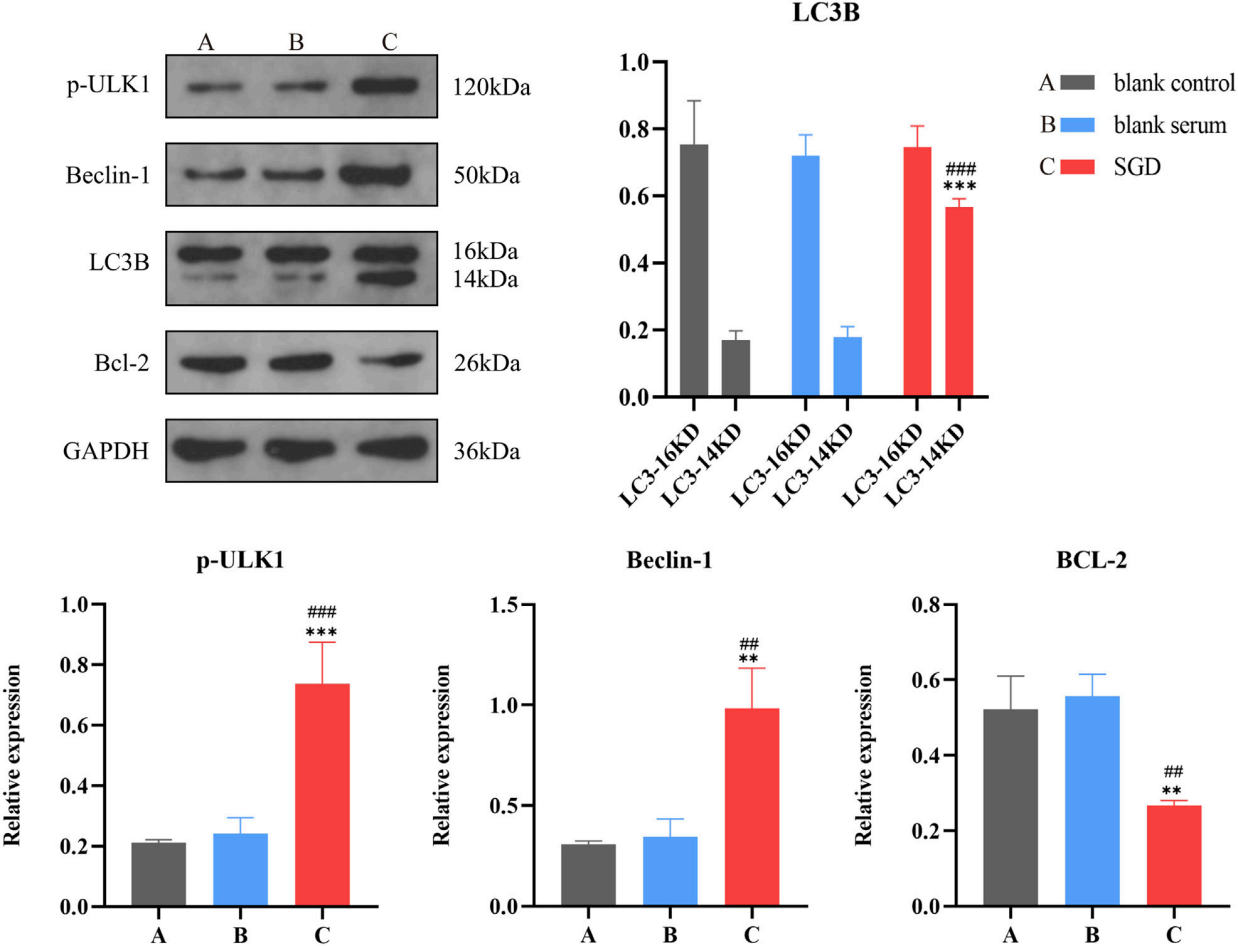
Effects of SGD on autophagy of EESCs. *, Compared with control group, *P* < 0.05, **P < 0.01, ***P < 0.001; #, compared with blank serum group, ##*P <* 0.01, ###*P* < 0.001.

Subsequently, we evaluated the expression of autophagy-related and apoptosis-related proteins among the three groups, as depicted in Figure 12. Compared to the control group and blank serum group, we observed a notable increase in the expression of autophagy-related proteins LC3B-II, p-UKL1, and Beclin-1 within the SGD-containing serum group (*P* < 0.01), while a marked reduction in Bcl-2 expression (*P* < 0.01). This result indicates that SGD can induce autophagy and exert a pro-apoptotic role in EESCs, thereby exerting an inhibitory effect on their activity.

## 4 Discussion

AM is a prevalent and challenging gynecological condition, with heavy menstrual bleeding, anemia, dysmenorrhea, and infertility as its common clinical manifestations (Bulun et al., 2023). As the disease progresses, the severity of menstrual pain tends to intensify, significantly disrupting patients’ daily lives and work while adversely affecting their overall health (Gruber and Mechsner, 2021). The study found that general health perceptions, as well as physical and societal functions, decline in AM patients. Thus, pain management of AM remains a primary focus of outpatient gynecological treatment (Siru, 2021). SGD, as an effective prescription for analgesia, is widely employed in the clinical management of various types of pain (Luo et al.,2023), particularly dysmenorrhea in women suffering from endometriosis and AM (Qu et al., 2020). Modern pharmacologic and clinical studies have demonstrated its efficacy in spasmolysis, analgesia, and anti-inflammatory effects (Xu et al., 2022). Ren and Jinli (2017) illustrated the effectiveness of SGD in alleviating clinical symptoms and reducing inflammatory markers among patients with dysmenorrhea. Previous studies conducted by our research group revealed that SGD can interfere with the survival of EESCs by inhibiting the Ras signaling pathway and disrupting Mitogen-Activated Protein Kinase (MAPK) signaling through estrogen receptor-mediated blockade, thereby affecting the progression of AM and symptom alleviation (Guan Y. et al., 2014).

Although the mechanistic processes and pathogenesis of AM remain unclear, several theories have been proposed to explain how the disease develops, including the invagination of the basal layer of the endometrium into the myometrium and increased invasion of the endometrium into the myometrium (Bulun et al., 2023). Recent studies have shown that endometriotic or adenomyotic stromal cells exhibit numerous epigenetic abnormalities, and these abnormalities relate to DNA methylation, histone modification, and miRNA expression (Bulun et al., 2023). Collectively, these findings indicate that the ectopic endometrium gains enhanced survival capabilities, which promote its invasion and proliferation in the myometrial tissue, thereby contributing to the development of AM.

MiR-21, one of the earliest discovered miRNAs, plays a pivotal role in cell growth, proliferation, and apoptosis in various tumors. Specifically, the upregulation of miR-21 has implications for tumor cell survival and distant metastasis (Tiong et al., 2023). In this study, we simultaneously detected the expression levels of miR-21 in both eutopic and ectopic endometria of patients with AM. The results showed that the expression level of miR-21 in the ectopic endometrium within the myometrium was significantly higher than that in the normal myometrium. Furthermore, autophagy was increased in EESCs following transfection with a miR-21 inhibitor. Thus, we consider miR-21 to be a key regulatory factor in the growth of AM lesions. Based on the qPCR assay results, we found that the relative expression of miR-21 was inhibited following SGD treatment. Moreover, PTEN expression was elevated following treatment with SGD. However, overexpressing miR-21 restored miR-21 levels after SGD treatment, thereby reversing the upregulation of PTEN expression. Wound healing assays and flow cytometry detection further confirmed that SGD can inhibit the proliferation of EESCs and promote their apoptosis by reducing the expression of miR-21. As a classic tumor suppressor gene, PTEN inhibits tumor cell proliferation and promotes tumor cell apoptosis by inhibiting numerous canonical signaling pathways, such as the PI3K/AKT pathway (Lee et al., 2018). Existing studies have confirmed that the miR-21-5p/PTEN regulatory axis is extensively involved in the occurrence and development of various cancers (Tang et al., 2018; Yan et al., 2020), and targeted inhibition of miR-21 expression has become a potential novel target in the field of tumor therapy (Jinhua et al., 2022). Herein, we reported similar results: SGD-induced inhibition of miR-21 expression may suppress the progression of AM. This effect may be associated with its regulation of the autophagic and apoptotic mechanisms in EESCs.

Autophagy and apoptosis play a critical role in endometrial growth cycles. Evidence shows that the reduced apoptosis of refluxed endometrial cells may improve their survival rate at ectopic sites, thereby promoting the development of endometriosis (Dmowski et al., 2001; Gebel et al., 1998). We observed that the expressions of LC3B and unc-51 like autophagy activating kinase 1 (ULK1) were markedly lower in both AM ectopic and eutopic endometria when compared to normal myometrium and endometrium samples, and a more pronounced decrease in Recombinant human Beclin-1 levels was noted specifically in the eutopic endometrium relative to all examined control groups. Beclin-1 and ULK1 are essential for initiating autophagy, and LC3B is a marker of autophagy that penetrates the entire autophagy process. Furthermore, an increased expression of Bcl-2 protein was detected in the AM ectopic endometrium. Bcl-2 plays a crucial role as an anti-apoptotic gene. Studies show that the reduction in PTEN activates the PI3K/AKT/mTOR signaling pathway, thereby leading to enhanced proliferation and invasion of endometrial cells (Zhang H. et al., 2024). mTOR-mediated suppression of autophagy plays a direct role in the pathogenesis of endometriosis (Choi et al., 2014; Choi et al., 2015; Glaviano et al., 2023). In our findings, the PI3K/AKT/mTOR pathway is aberrantly activated in the ectopic endometrium of AM. This activation contributes to the inhibition of autophagy and reduced apoptosis of active EESCs within these lesions, ultimately leading to their uncontrolled proliferation.

The induction of autophagy exerts a proapoptotic effect on normal human endometrial cells (Choi et al., 2012). Beibei et al. (2023) reveals that SGD inhibits the PI3K/AKT/mTOR pathway while enhancing autophagy in dopaminergic neurons within a rat model of Parkinson’s disease induced by 6-hydroxydopamine. In the present study, we observed a decrease in phosphorylated AKT and mTOR in EESCs after culturing with SGD-containing serum. These changes were associated with a reduction in autophagy-related proteins LC3B-II, p-ULK1, and Beclin-1, alongside an increase in the anti-apoptotic protein Bcl-2 in EESCs. In the previous studies of our research group, it was found that the upregulation of miR-21 results in diminished PTEN expression, which subsequently leads to abnormal activation of the PI3K/AKT/mTOR pathway, which is involved in aberrant autophagy in EESCs (Jia et al., 2025). Therefore, we further hypothesize that SGD can promote autophagy and apoptosis in EESCs by downregulating the expression of miR-21, and this process is associated with the inhibition of the PI3K/AKT/mTOR signaling pathway. Notably, in the present study, we observed that SGD specifically reduced the levels of p-AKT and p-mTOR without affecting the total expression of PI3K. This seemingly contradictory phenomenon actually reveals the mechanism of action of SGD. Our data showed that SGD can effectively downregulate the highly expressed miR-21 in EESCs. It is well-established that miR-21 plays a critical role in tumors and other hyperplastic diseases by targeting multiple tumor suppressor genes (Surina et al., 2021; Wang et al., 2020). Among these targets, one of the most well-characterized target is PTEN—a core negative regulator of the PI3K/AKT/mTOR signaling pathway (Tsai et al., 2017; Zhang H.-P. et al., 2024). PTEN terminates the transduction of downstream signals by dephosphorylating PIP3, the product of PI3K (Csolle et al., 2020). Therefore, we propose the following core mechanism to explain all the findings of this study: SGD effectively reduces the expression of miR-21, thereby relieving its inhibition of PTEN and restoring PTEN expression. The upregulated PTEN further enhances the degradation of Phosphatidylinositol 3,4,5-trisphosphate (PIP3); even though the total protein level of PI3K remains unchanged, the downstream activation signals generated by PI3K are significantly attenuated. Ultimately, this leads to a decrease in the levels of p-AKT and p-mTOR. In future studies, we will further verify the key links of this mechanism through supplementary experiments. For instance, we will use the dual-luciferase reporter gene assay to determine whether miR-21 and its target PTEN are required for the SGD-regulated signaling pathway.

This report has afforded a new potential mechanism by which SGD regulates the growth of EESCs, but the limitations of this study should not be ignored. First, although the *in vitro* EESCs model is the most widely used and highly recognized model in current studies on the cellular mechanisms of AM, this study has a limitation in the lack of *in vivo* validation data from animal models. We have included AM animal model experiments in our subsequent research plan to further verify the *in vivo* applicability of the *in vitro* study findings. Second, due to the constraints of the current research status and the design of this study, patient serum samples were not included to detect the expression of miR-21; however, the levels of miRNAs in tissues and circulation do not always show a parallel trend, which may result in certain limitations in the reference value of the study conclusions for clinical translation. Finally, given that miR-21 plays a crucial role in tissue repair and immune regulation, systemic inhibition of miR-21 may induce potential off-target effects or unintended apoptosis, and this point should be emphasized as a limitation of this study.

## 5 Conclussion

In conclusion, the present study explored the autophagy inhibition in AM ectopic lesions, which is associated with the progression of AM. SGD can induce autophagy and promote apoptosis, thereby debilitating the proliferation and progression of EESCs by inhibiting miR-21 expression, with PTEN as the target. This process affects the activity of the PI3K/AKT/mTOR pathway.

## Data Availability

The raw data supporting the conclusions of this article will be made available by the authors, without undue reservation.

## Abbreviations

SGD, Shaoyao Gancao Decoction; AM, Adenomyosis; CIN3, Cervical Intraepithelial Neoplasia Grade 3; EESCs, Ectopic endometrial stromal cells; RT-qPCR, Reverse Transcription-Quantitative Polymerase Chain Reaction; WB, Western blotting; MTT, 3-(4,5-Dimethylthiazol-2-yl)-2,5-diphenyltetrazolium bromide assay; miR-21, MicroRNA-21; PTEN, Phosphatase and tensin homolog; PI3K, Phosphatidylinositol 3-kinase; AKT, Protein kinase B; mTOR, Mammalian target of rapamycin; TCM, Traditional Chinese Medicine; Baishao, Radix Paeoniae Alba; Zhigancao, Glycyrrhiza uralensis Fisch; ICC, Immunocytochemistry; FCM, Flow cytometry; TEM, Transmission electron microscopy; NC, Negative control; FBS, Fetal bovine serum; SDS-PAGE, Sulfate-polyacrylamide gel electrophoresis; LSD, Least significant difference; Beclin-1, Beclin 1 protein; ULK1, Unc-51 like autophagy activating kinase 1; LC3, Light chain 3; Bcl-2, B-cell lymphoma-2; miRNAs, MicroRNAs; MAPK, Mitogen-Activated Protein Kinase; PIP3, Phosphatidylinositol 3,4,5-trisphosphate.
