# Supplementary material for: Shaoyao gancao decoction induces autophagy by modulating miR-21 and inhibiting the AKT/mTOR signaling pathway in adenomyosis-derived ectopic endometrial stromal cells
Source: Front Pharmacol. 2025 Nov 27;16:1665911. doi: 10.3389/fphar.2025.1665911 (PMC12695794; doi:10.3389/fphar.2025.1665911)
Supplement: Supplementary file 1 [file Supplementaryfile1.docx]

Supplementary Material

# Supplementary Figures and Tables

## Supplementary Figures

Supplementary Figure 1：The western blot strips of p-UKL1 among tissues


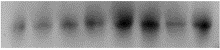
112kDa

Supplementary Figure 2：The western blot strips of LC3B among tissues


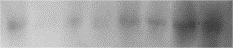
15kDa

Supplementary Figure 3：The western blot strips of Beclin-1 among tissues


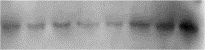
60kDa

Supplementary Figure 4：The western blot strips of Bcl-2 among tissues


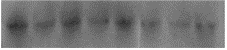
26kDa

Supplementary Figure 5：The western blot strips of DAPDH among tissues


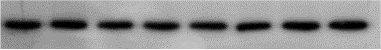
36kDa

Supplementary Figure 6：The western blot strips of PTEN among tissues


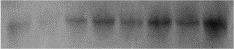
54kDa

Supplementary Figure 7：The western blot strips of PI3K among tissues


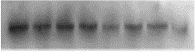
123kDa

Supplementary Figure 8：The western blot strips of AKT among tissues


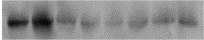
60kDa

Supplementary Figure 9：The western blot strips of p-AKT among tissues


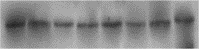
60kDa

Supplementary Figure 10：The western blot strips of mTOR among tissues


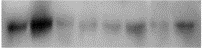
250kDa

Supplementary Figure 11：The western blot strips of p-mTOR among tissues


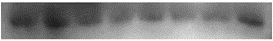
289kDa

Supplementary Figure 12：The western blot strips of PTEN among cells


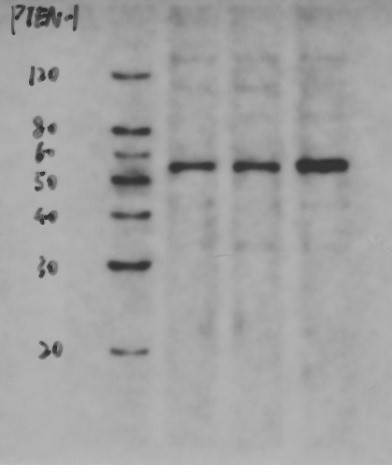
54kDa

Supplementary Figure 13：The western blot strips of PI3K among cells


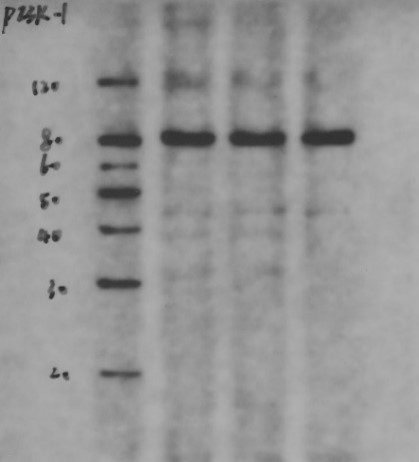
80kDa

Supplementary Figure 14：The western blot strips of AKT among cells


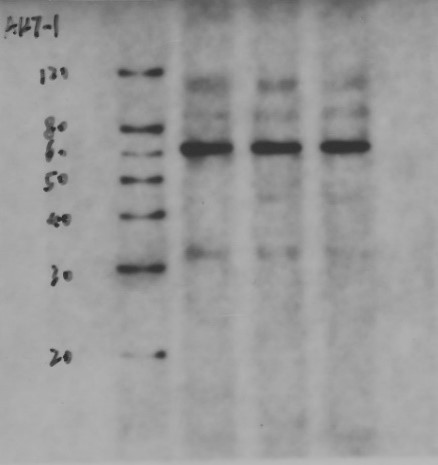
60kDa

Supplementary Figure 15：The western blot strips of p-AKT among cells


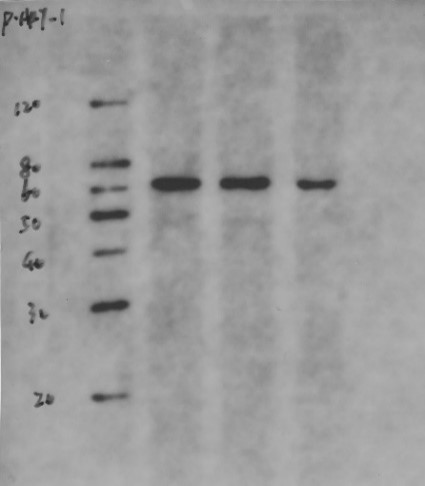
60kDa

Supplementary Figure 16：The western blot strips of mTOR among cells


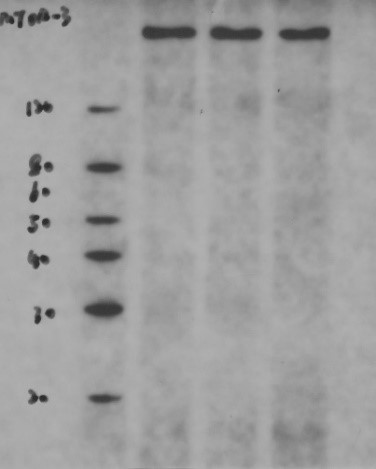
250kDa

Supplementary Figure 17：The western blot strips of p-mTOR among cells


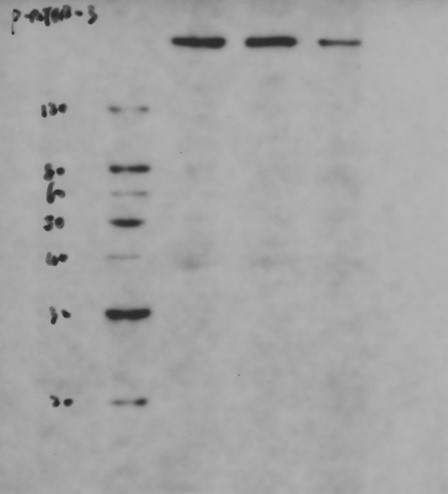
289kDa

Supplementary Figure 18：The western blot strips of GAPDH among cells


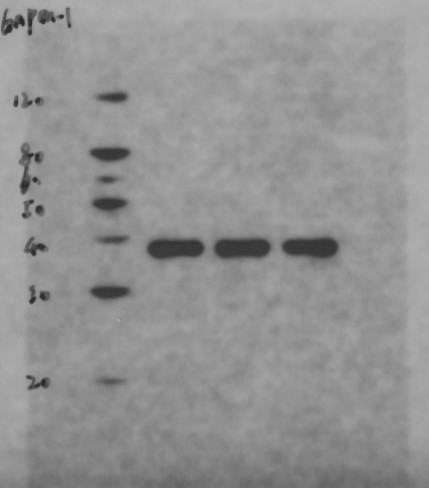
36kDa

Supplementary Figure 19：The western blot strips of Bcl-2 among cells


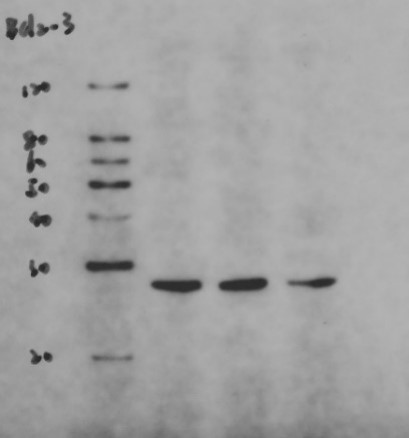


Supplementary Figure 20：The western blot strips of p-ULK1 among cells


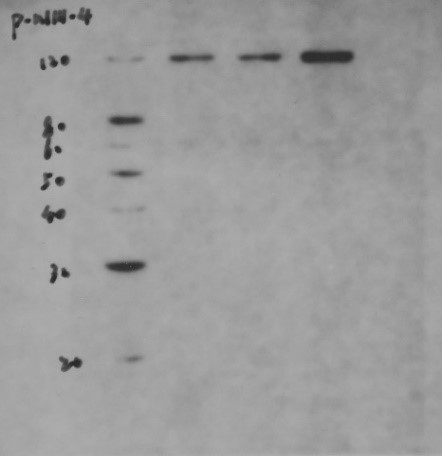


Supplementary Figure 21：The western blot strips of Beclin-1 among cells


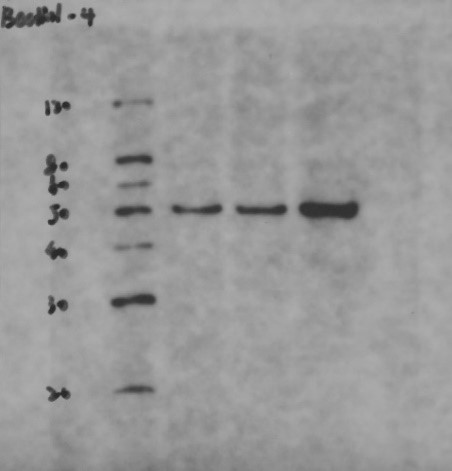


Supplementary Figure 22：The western blot strips of LC3B among cells


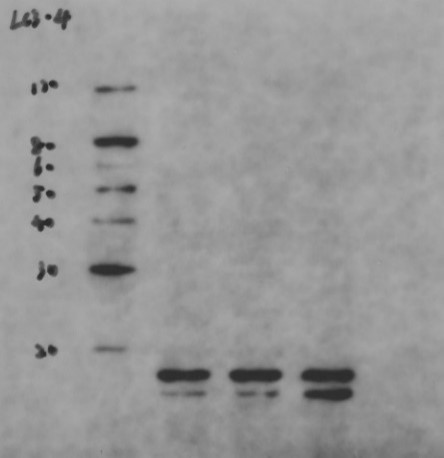


Supplementary Figure 23：The western blot strips of DAPDH among cells


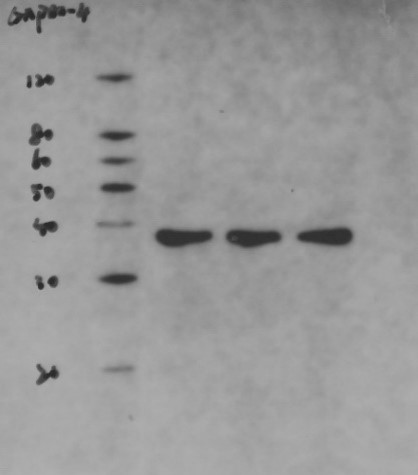


## Supplementary Tables

**Supplementary Table 1：Primer sequences of RT-PCR (mRNA)**

| Gene | Forward Primer  （5’→ 3’） | Reverse primer  （3’→ 5’） |
| --- | --- | --- |
| miR-21 | GCTGTGGTAGCTTATCAGACTG | GTGCAGGGTCCGAGGT |
| PI3K | AAGGCCACTGTGGTTGAATTG | TCTTGCCGTAAATCATCCCC |
| AKT | CTTCTCTGTGGCGCAGTGC | ACATGGAAGGTGCGTTCGAT |
| mTOR | TTCTGTGGCTGTGAGGTCTGA | GCCTTCTGCCTCTTATGGGC |
| GAPDH | GATTCCACCCATGGCAAATT | TCTCGCTCCTGGAAGATGGT |

**Supplementary Table 2：Primer sequences of RT-PCR**

| Gene | Forward Primer  （5’→ 3’） | Reverse primer  （3’→ 5’） |
| --- | --- | --- |
| PTEN | ACTATTCCCAGTCAGAGGCG | GAACTTGTCTTCCCGTCGTG |
| hsa-miR-21-5p | GTCGTATCCAGTGCAGGGTCCGAGGTA  TTCGCACTGGATACGACTCAACATC | TGCGCTAGCTTATCAGACTGAT |
| GAPDH | TCAAGAAGGTGGTGAAGCAGG | TCAAAGGTGGAGGAGTGGGT |
