# Supplementary material for: Shaoyao gancao decoction induces autophagy by modulating miR-21 and inhibiting the AKT/mTOR signaling pathway in adenomyosis-derived ectopic endometrial stromal cells
Source: Front Pharmacol. 2025 Nov 27;16:1665911. doi: 10.3389/fphar.2025.1665911 (PMC12695794; doi:10.3389/fphar.2025.1665911)
Supplement: Supplementary file 2 [file Supplementaryfile2.pdf]

Supplementary Table 3

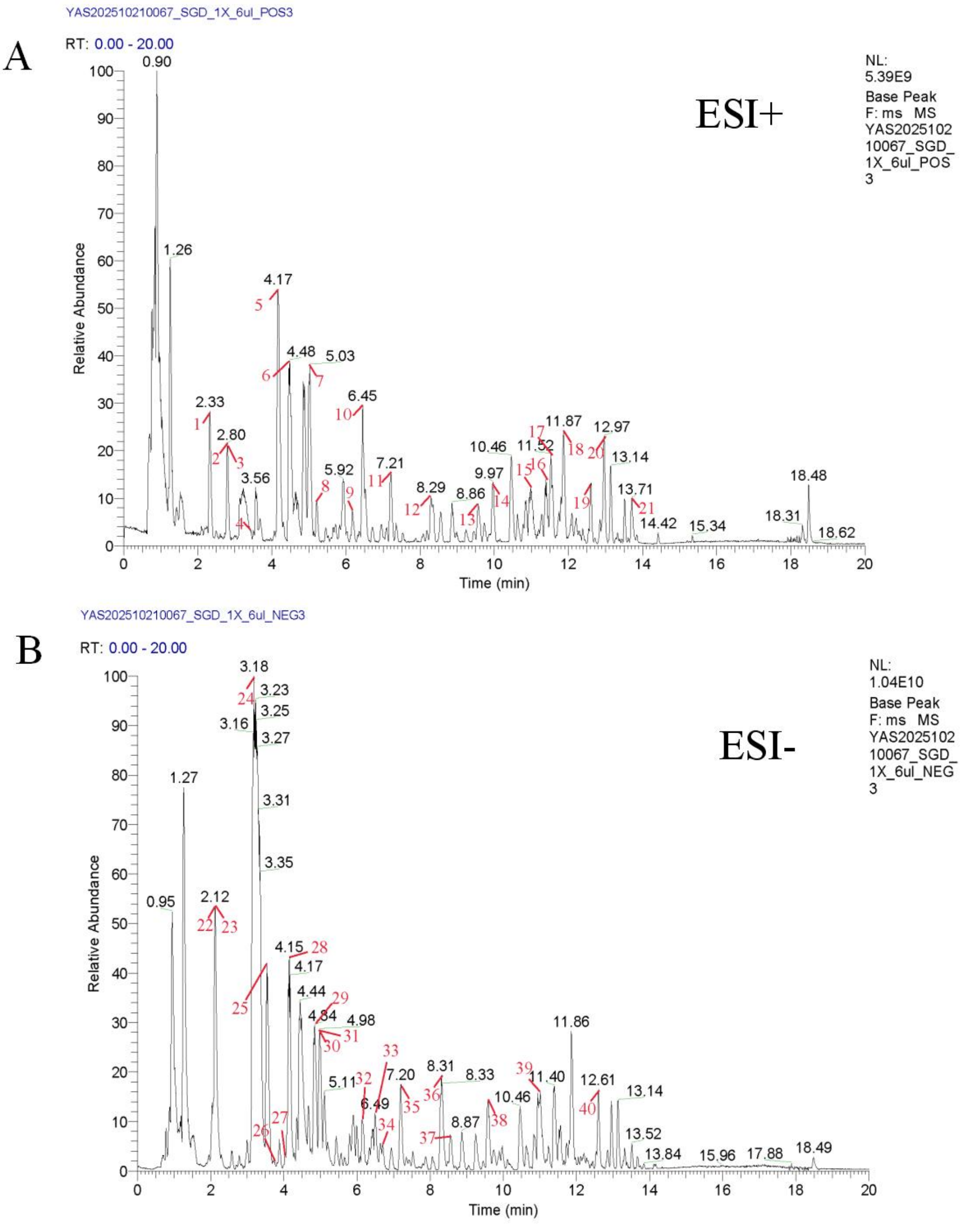

Fig.1. UPLC-HRMS identification results of the main chemical components of SGD. (A) BPC of SGD in positive ion mode; (B) BPC of SGD in negative ion mode.

Table 3 Identification of main chemical constituents in extracts from SGD

PM	RT/min	Molecular formula	Adduct	Error (ppm)	m/z	Compound name
1	2.3	$C_{14}H_{20}N_2O_3$	$[M+H-C_5H_9NO]^+$	1.1	166.0862	Valylphenylalanine

2	2.77	C14H28ClNO	[M+H]+	7.9	262.1911	2-Chloro-N-dodecylacetamide
3	2.83	C7H6O2	[M+H]+	2.5	123.044	Benzoic acid
4	3.4	C23H28O12	[M+NH4]+	2.5	514.1915	Oxypaeoniflorin
5	4.14	C23H28O11	[M+H]+	0.2	481.17	Albiflorin
6	4.44	C10H13NO3	[M+H-NH3]+	1.1	179.0701	.beta.-Homotyrosine
7	4.99	C15H12O4	[M+H]+	0	257.0805	Liquiritigenin
8	5.18	C17H23NO8	[M+H-C6H12O5]+	0.5	206.0811	3-C-(((2-(3-Carboxypropanamido)benzyl)oxy)carbonyl)-1,5-dideoxypentitol
9	6.16	C26H30O13	[M+H]+	1.5	551.1753	4-(7-Hydroxy-4-oxo-3,4-dihydro-2H-chromen-2-yl)phenyl 2-O-(3,4-dihydroxy-4-(hydroxymethyl)tetrahydrofuran-2-yl)hexopyranoside
10	6.43	C22H22O9	[M+H]+	0.9	431.1333	Ononin
11	7.19	C15H12O4	[M+H]+	1.7	257.0797	Isoliquiritigen
12	8.29	C30H32O12	[M+NH4]+	0.1	602.2231	Benzoylpaeoniflorin
13	9.56	C30H44O4	[M+H]+	0.5	469.3309	Glabrolide
14	9.96	C16H12O4	[M+H]+	0.2	269.0806	Formononetin
15	11	C42H62O16.2NH3	[M+H]+	1.2	823.4102	Diammonium glycyrrhizinate
16	11.39	C42H62O16	[M+H]+	0.3	823.41	Glycyrrhizin
17	11.52	C21H24O5	[M+H]+	2	357.1691	Glyasperin C
18	11.86	C26H30O11	[M+H-C6H12O5]+	0.5	355.1176	Phellamurin
19	12.6	C20H16O6	[M+H]+	0.8	353.1017	Parvisoflavone B
20	12.95	C22H26O5	[M+H]+	1.5	371.1849	Glyasperin D
21	13.71	C26H42O4	[M+H-C18H3	0.2	149.0233	Di(2,6-dimethyl-4-heptyl) phthalate

			8O]+			
22	2.12	C7H6O5	[M-H]-	0.2	169.0139	Gallic acid
23	2.12	C18H24O12	[M-H-C12H18O9]-	2.6	125.0241	Licoagroside B
24	3.2	C23H28O13S	[M-H]-	3.9	543.1201	Ncgc00380481-01_c23h28o13s_
25	3.54	C9H10O3	[M-H]-	0.1	165.0557	Benzenepropanoic acid, 4-hydroxy-
26	3.73	C8H8O5	[M-H]-	1.6	183.0302	4-O-Methylgallic acid
27	4.04	C15H14O6	[M+HCOO]-	5.1	335.0789	Catechin
28	4.14	C23H28O11	[M+HCO2]-	4.1	525.1635	Albiflorin
29	4.84	C26H30O13	[M-H]-	4.3	549.1639	Liguiritigenin-7-O-beta-D-apiosyl-4'-O-beta-D-glucoside
30	4.98	C21H22O9	[M-H]-	4.7	417.1209	Liquiritin
31	5.04	C41H32O26	[M-2H]2-	4.6	469.0539	1,2,3,4,6-Pentagalloyl .beta.-D-glucose
32	6.16	C26H30O13	[M+Cl]-	5.5	585.1411	Neolicuroside
33	6.49	C21H22O9	[M-H]-	5.7	417.121	Isoliquiritin
34	6.62	C6H6O	[M-H]-	6.8	93.0343	Phenol
35	7.17	C15H12O4	[M-H]-	4.4	255.0671	Isoliquiritigenin
36	8.26	C30H32O12	[M+FA-H]-	5.6	629.1905	NCGC00169221-01
37	8.51	C42H62O18	[M-H]-	4.4	853.3899	(3.beta.,9.xi.)-3-((6-Deoxyhexopyranosyl-(1->2)hexopyranosyl-(1->3)hexopyranosyl)oxy)-14-hydroxybufa-4,20,22-trienolide
38	9.53	C42H62O17	[M-H]-	4.9	837.3949	Licoricesaponin g2
39	10.98	C42H62O16	[M-H]-	4.9	821.4002	Licoricesaponin h2
40	12.59	C20H16O6	[M-H]-	2.9	351.089	Ncgc00169972-02!3,6-dihydroxy-8-methoxy-3-methyl-2,4-dihydrobenzo[a]anthracene-1,7,12-trione
